# Novel and efficient synthesis of 5-chloro-6-methoxy-3-(2-((1-(aryl)-1*H*-1,2,3-triazol-4-yl)methoxy)ethyl)benzo[*d*]isoxazole derivatives as new *α*-glucosidase inhibitors

**DOI:** 10.1016/j.bbrep.2025.102074

**Published:** 2025-06-05

**Authors:** Ram Reddy Mudireddy, Rambabu Gundla, Chandra Prakash Koraboina, Vani Madhuri Velavalapalli, Venkata Veernjaneya Sarma Dhulipalla, Gowri Sankararao Burle, Sreekantha B. Jonnalagadda, Naresh Kumar Katari

**Affiliations:** a Department of Chemistry, School of Science, GITAM (Deemed to be University), Hyderabad, Telangana, 502 329, India; b B.V.Raju Institute of Technology, Vishnupur, Medak Dist, Narsapur, Telangana, 502313, India; c Analytical R&D, Catalent Pharma Solutions, 2725 Scherer Drive, St. Petersburg, FL, 33716, USA; d Research and Development, HIKMA Pharmaceuticals, 1809 N Wilson Rd, Columbus, OH, 43228, USA; e School of Chemistry & Physics, College of Agriculture, Engineering & Science, Westville Campus, University of KwaZulu-Natal, P Bag X 54001, Durban, 4000, South Africa

**Keywords:** Benzisoxazole, 1,2,3-Triazole, Diethyl ether, Copper iodide, THF, Synthesis

## Abstract

A new series of benzisoxazole derivatives (**9a-o**) were designed by using molecular hybridization approach and synthesized *via* click-chemistry. All the synthesized compounds were evaluated for their α-glucosidase enzyme inhibition and antibacterial activity. All tested compounds (**9a-o**) exhibited a promising α-glucosidase inhibitory activity with IC_50_ range of 14.69–38.71 nmol in comparison with the positive drug **Acarbose** (IC_50_ 35.91 nmol). Additionally, these compounds have found to be active against *B. cereus* and *E. coli*. The *in vitro* inhibition results supported to *in silico*. Additionally, the compounds were subjected to computational drug-likeness/ADME testing, which revealed that this all the compounds had good ADME profiles in addition to exhibiting drug-like qualities. SAR indicates that analysis revealed that electron-withdrawing substituents such as Br and CF_3_ at specific positions significantly enhanced α-glucosidase inhibition, while unsubstituted and ortho-methoxy phenyl derivatives also showed potent activity, highlighting the benzo[d]isoxazole–triazole scaffold as a promising pharmacophore for developing novel anti-diabetic agents.

## Highlights

• Novel benzo[d]isoxazole-triazole hybrids were synthesized as α-glucosidase inhibitors.
• All compounds were prepared via Cu(I)-catalyzed click chemistry in good yields.
• Compounds **9a** and **9j** showed top docking scores of −9.2 and −9.4 kcal/mol, respectively.
• All derivatives showed potent *α*-glucosidase inhibition with IC_50_ range of 14.69–38.71 nmol.
• Compounds **9f**, **9h**, and **9l** showed antibacterial activity against *Bacillus* *cereus.*

## 1 Introduction

Diabetes mellitus (DM) refers to a group of chronic metabolic disorders marked by hyperglycemia resulting from dysfunctions in insulin action, synthesis, or both [1,2]. Common forms of diabetes mellitus can lead to macrovascular complications such as ischaemic heart disease, stroke, and peripheral vascular disease, as well as microvascular complications including retinopathy, nephropathy, and neuropathy, resulting in multisystem effects [[3], [4], [5]]. In 2022, the worldwide number of diabetic patients was 462 million, and it is projected to reach 578 million by 2030 [6,7]. The World Health Organisation (WHO) projected an increase to 642 million cases of diabetes mellitus (DM) globally by 2040 [8]. The most frequent method of treating diabetes is by blocking the carbohydrase enzyme α-glucosidase, which decreases the absorption of glucose [9,10]. The α-glucosidase enzyme, which is functionally identical to α-amylase, catalyses the hydrolytic activity that releases glucose molecules from the carbohydrates [11,12]. Inhibitors of α-amylase and α-glucosidase, such as **acarbose**, **voglibose**, and **miglitol**, decrease the risk of developing type-2 diabetes (T2DM) [13]. Despite their rapid onset of action and potent therapeutic effects, oral diabetes drugs may induce adverse side effects. The main issue with these drugs is that they only alleviate symptoms of diabetes, not the underlying pathogenesis. Therefore, it is of the highest priority to develop effective, safe, and non-toxic alternatives for the treatment of T2DM.

**Fig. 1:**
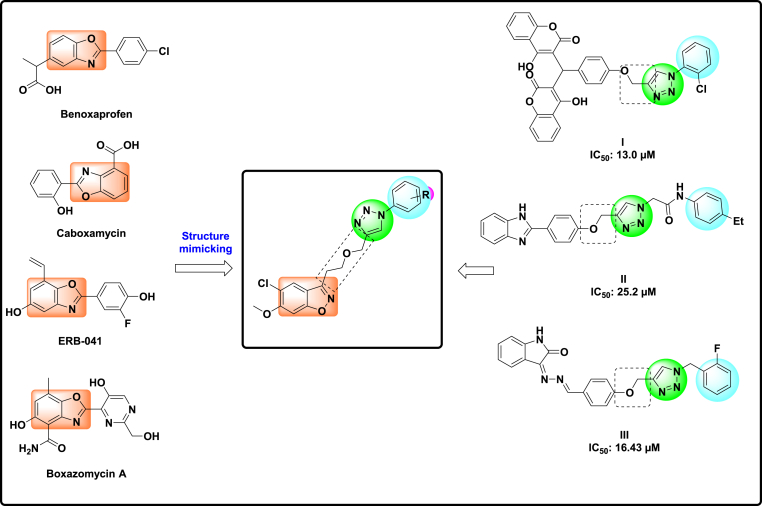
Design strategy for the new benzo[*d*]isoxazole-triazole derivatives (**9a-o**).

*N*-Heterocycles are an important class of heterocycles extensively employed in the development of novel bioactive compounds. Benzoxazole is a building block present in several natural compounds, as well as in a significant number of commercially marketed pharmaceuticals, including **Benoxaprofen**, **Caboxamycin**, **ERB-041**, and **Boxazomycin** A (Fig. 1) [14,15]. Specifically, aryl-substituted benzoxazole derivatives hold significant importance in pharmacology owing to their diverse applications, which encompass anti-tumor [16,17], anti-fungal [18], anti-bacterial [19,20], anti-HIV [21], anti-tubercular [22,23] and anti-inflammatory [24] properties.

On the other hand, 1,2,3-triazole and its derivatives are essential frameworks exhibiting diverse pharmacological properties in drug discovery [[25], [26], [27], [28], [29],^53^,^54^]. Recent research has identified 1,2,3-triazoles as effective anti-α-glucosidase drugs [[30], [31], [32]], including compound **I** (Fig. 1), which serves as a powerful competitive inhibitor of α-glucosidase [33]. The derivatives demonstrated IC_50_ values between 13.0 and 75.5 μM. A series of benzimidazole-1,2,3-triazoles with a phenoxy linker (**II**) were systematically constructed as α-glucosidase inhibitors and demonstrated substantial inhibitory action in comparison to their parent compounds and the positive control, acarbose [34]. Recently, a series of indolinone-substituted phenoxy-methyltriazole derivatives were synthesized, with derivative **III** being the most active and exhibiting competitive inhibition of the enzyme [35]. *In silico* evaluations verified that the phenoxy-1,2,3-triazole moiety enhances compound stability *via* hydrogen bonding and pi-alkyl interactions.

Based on the literature search here we designed and synthesized a new class of benzo[*d*]isoxazole-triazole derivatives as promising *α*-glucosidase inhibitors. *In silico* evaluations were conducted on develop new derivatives. We conducted the 1,3-dipolar cycloaddition under catalytic circumstances to synthesize the final targeted products with a good to excellent yields. Consequently, the biological activity of these compounds may eventually result in promising enzymatic inhibition as well as antibacterial effectiveness.

## 2 Results and discussion

### 2.1 Docking studies

**Fig. 2:**
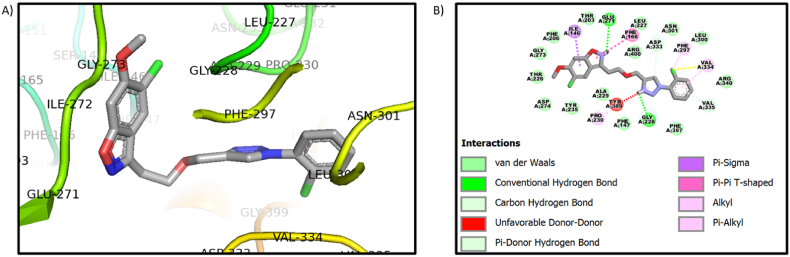
A) Image of the compound **9a** in the pocket of 3WY1 B) interaction image of compound **9a** with Protein ID 3WY1.

**Fig. 3:**
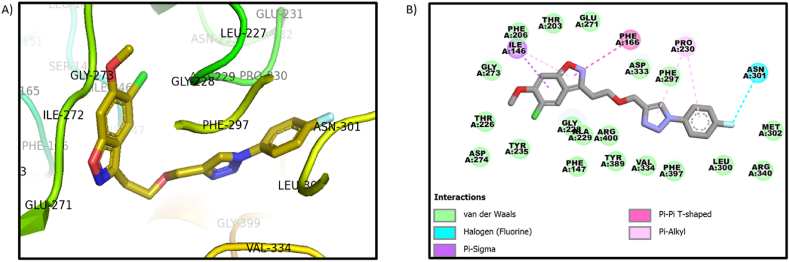
A) Image of the compound **9j** in the pocket of 3WY1 B) interaction image of compound **9j** with Protein ID 3WY1.

*In silico* investigations are carried out using starting from a known target structure to find possible ligands. The highest docking score molecules are synthesized and evaluated for their potency. The interaction images of the compound **9a** and **9j** with protein PDB ID 3WY1 are shown in Fig. 2, Fig. 3. The docking scores are given in Table 1. One of the crucial residues for linkage specificity was thought to be THR 203 or ALA are typically found at this location in specific glucosidases. The homologues of HaG share six conserved residues: Asp62, Arg400, Phe166, Thr203, Phe206, and Phe147. Additionally, Gly228 is only located in HaG[36].

**Table 1:** Molecular docking binding energies of designed compounds (**9a-o**).

Compound	Ar	Docking Scores (Kcal/Mol)
**9a**	Ph	−9.2
**9b**	2-ClC_6_H_4_	−8.9
**9c**	3-ClC_6_H_4_	−9.0
**9d**	4-ClC_6_H_4_	−8.9
**9e**	2-F-3-H_3_C–C_6_H_3_	−9.0
**9f**	2-H_3_CC_6_H_4_	−8.8
**9g**	2-H_3_CO–C_6_H_4_	−8.6
**9h**	4-H_3_CO–C_6_H_4_	−8.7
**9i**	2-F-3-Br-C_6_H_3_	−9.0
**9j**	4-FC_6_H_4_	−9.4
**9k**	2-F_3_C–C_6_H_4_	−9.0
**9l**	4-F_3_C–C_6_H_4_	−9.1
**9m**	3.4-diMeC_6_H_3_	−9.1
**9n**	4-BrC_6_H_4_	−9.3
**9o**	3-F_3_C-4-Br-C_6_H_3_	−9.2
**Co-Crystal**	PRU	−7.7
**Acarbose**		−7.8

Compound **9a** showed a conventional hydrogen bond with the crucial amino acids GLY-228 and GLU-271. The benzene ring of benzisoxazole showed Pi-sigma interaction with ILE-146. The Pi-Pi interaction is shown with PHE-166. The alkyl and Pi-alkyl interaction is shown with the PHE-166, PHE-297, VAL-334, and PRO-230 amino acids. The carbon-hydrogen interaction is shown with the amino acids ASP-333 and VAL-335. The amino acids THR-203, PHE-206, GLY-273, THR-226, ASP-274, TYR-235, PHE-147, ASN-301, LEU-227, LEU-300 and PHE-397 showed van der Waals interaction. In order to discover more precise and stable binding conformations, the unfavorable donor-donor penalty is a strategy used in molecular docking scoring functions to discourage poses when many hydrogen bond donors on the ligand are driven into unfavorable interactions with the protein. In 9a compound interaction amino acid TYR-389 showed unfavorable donor donor interaction.

**Fig. 4:**
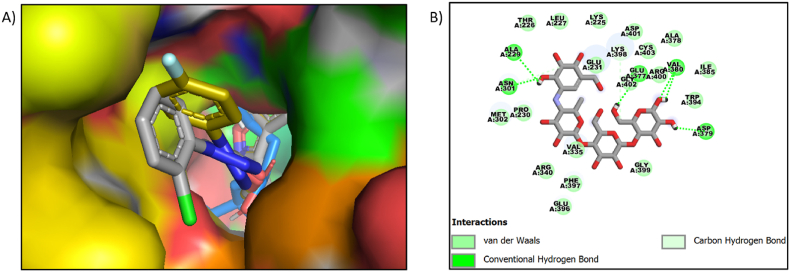
A) The Surface image of compound **9a** (grey color), 9j (yellow), and cocrystal (blue) in the same pocket. B) Acarbose interactions with protein 3WY1.

The benzisoxazole derivative compound **9j** showed van der Waals interactions with amino acids PHE-206, THR-203, GLU-271, GLY-228, ALA-229, ARG-400, ASP-333, PHE-297, TYR-389, VAL-334. The fluorine atom of the compound **9j** showed halogen interaction with the amino acids ASN-301. The Pi-sigma interaction is seen with the amino acid ILE-146 with the phenyl group of the benzisoxazole. The Pi-Pi interaction is shown with the amino acid PHE-166. The amino acid PRO-230 showed interaction with benzene and 5-membered rings. The interaction image of the compound **9j** with protein PDB ID 3WY1 is shown in Fig. 3. Two compounds, **9a and 9j,** interacted well with the crucial amino acids THR-203 GLY-228. AND PHE-297. The acarbose interactions were shown in Fig. 4(B). Fig. 4 (A) shows the surface image of all the three compounds (**co-crystal, 9a, 9j**).

The docking analysis of benzisoxazole derivatives **9a** and **9j** highlights the critical role of electronic and steric effects in mediating their interactions within the enzyme's active site. Compound **9a** demonstrated strong binding affinity through conventional hydrogen bonds with key residues GLY-228 and GLU-271, facilitated by polar functional groups acting as hydrogen bond donors or acceptors. The aromatic benzisoxazole moiety further contributed to π-sigma and π-π stacking interactions with ILE-146 and PHE-166, respectively, enhancing electronic complementarity. Additionally, hydrophobic alkyl and π-alkyl interactions with PHE-166, PHE-297, VAL-334, and PRO-230, along with carbon–hydrogen bonding and widespread van der Waals contacts, suggest that compound **9a** is sterically well-accommodated in the binding pocket. In contrast, compound **9j** exhibited notable halogen bonding between its fluorine substituent and ASN-301, a highly directional electronic interaction that enhances specificity. Although **9j** retained important π-sigma and π-π interactions similar to **9a**, it showed fewer hydrophobic contacts, indicating a slightly less bulky or hydrophobic profile. Nevertheless, its interactions with PRO-230 and multiple residues *via* van der Waals forces affirm a good steric fit. Overall, compound **9a** benefits from a combination of strong hydrogen bonding and hydrophobic interactions, whereas compound **9j** introduces halogen bonding as a unique contributor to binding affinity, underscoring how subtle changes in electronic and steric properties can significantly influence inhibitor enzyme interactions.

### 2.2 Design and assessment of physicochemical characteristics

**Fig. 5:**
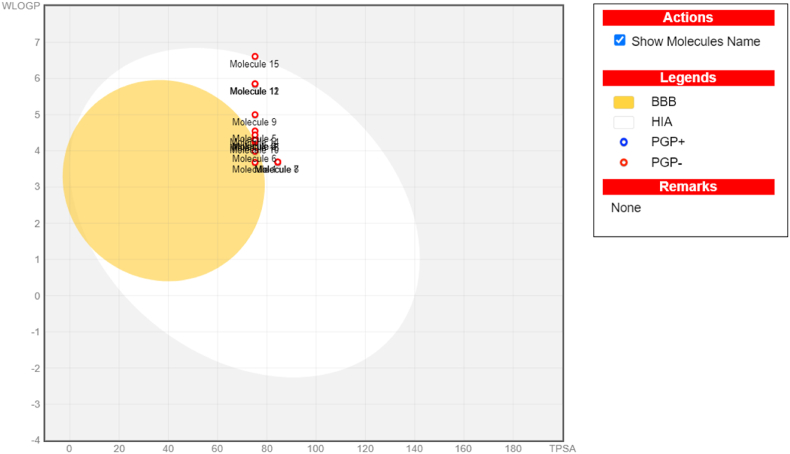
BOILEDEgg of benzisoxazole derivatives.

Physical properties play an important role in determining a molecule's efficacy and therapeutic potential during drug development [37]. Several critical criteria must be evaluated when performing computational analysis of structural and physicochemical properties. One typical strategy is to employ topological fingerprints, which take into account all molecular fragments that follow linear paths up to a certain number of links. This method makes it easier to calculate key physicochemical parameters such as total polar surface area (TPSA), molar refractivity (MR), hydrogen bond acceptors (HBA), hydrogen bond donors (HBD), rotatable bonds (RB), heavy atoms (HA), and heavy aromatic atoms (HAA). These properties were computed using the SwissADME server. The analytical results, which are in line with accepted standards for blood-brain barrier (BBB) permeability and predictive modeling, are compiled in Table 2. Research has indicated that medications with reduced molecular weight and lipophilicity have better transcellular and paracellular absorption and clearance, which leads to moderate toxicity and greater renal excretion [38]. Lipinski's "Rule of Five," which states that a chemical is considered drug-like if it satisfies the following requirements, is commonly used to evaluate drug-like molecules (DLMs): a molecular weight of less than 500 Da, a log P value of less than 5, no more than five hydrogen bond donors, and no more than ten hydrogen bond acceptors [39]. The Veber rule suggests that compounds with 140, 10, or 12 hydrogen bond donors, acceptors, and polar surface areas have higher oral bioavailability [40]. All of the compounds have zero Veber violations. All benzisoxazole substances have anticipated pharmacokinetic properties, such as brain-blood barrier (BBB) and gastrointestinal absorption (GIA). The Brain or IntestinaLEstimateD Permeation technique (BOILEDEgg) was visually illustrated by calculating the lipophilicity (WLOGP vs TPSA) (Fig. 5) [41]. The projection shows that the yellow ellipse contains two molecules, whereas the white ellipse contains all of the other molecules. This implies that compounds may have superior GIA properties but inferior BBB properties. All medicines with efflux activity predicted by P-glycoprotein (PGP) in the central nervous system (Table 2).

**Table 2:** Physio-chemical properties of compounds **9a-9o.**

Compound	MW (molecular weight)	TPSA (total polar surface area)	iLOGP	BBB permeant	Lipinski #violations	MR (molar refractivity)	# Hydrogen bond acceptor	#Hydrogen bond donor
**9a**	**384.82**	**75.2**	**3.82**	**Yes**	**0**	**100.58**	**6**	**0**
**9b**	419.26	75.2	3.88	No	0	105.59	6	0
**9c**	419.26	75.2	3.99	No	0	105.59	6	0
**9d**	419.26	75.2	3.95	No	0	105.59	6	0
**9e**	416.83	75.2	3.95	No	0	105.5	7	0
**9f**	398.84	75.2	3.96	Yes	0	105.55	6	0
**9g**	414.84	84.43	3.96	No	0	107.07	7	0
**9h**	414.84	84.43	3.96	No	0	107.07	7	0
**9i**	481.7	75.2	4	No	0	108.24	7	0
**9j**	**402.81**	**75.2**	**3.84**	**Yes**	**0**	**100.54**	**7**	**0**
**9k**	452.81	75.2	4.03	No	0	105.58	9	0
**9l**	452.81	75.2	4.06	No	0	105.58	9	0
**9m**	412.87	75.2	3.88	No	0	110.51	6	0
**9n**	463.71	75.2	4.06	No	0	108.28	6	0
**9o**	531.71	75.2	4.22	No	1	113.28	9	0

### 2.3 Chemistry

**Scheme 1:**
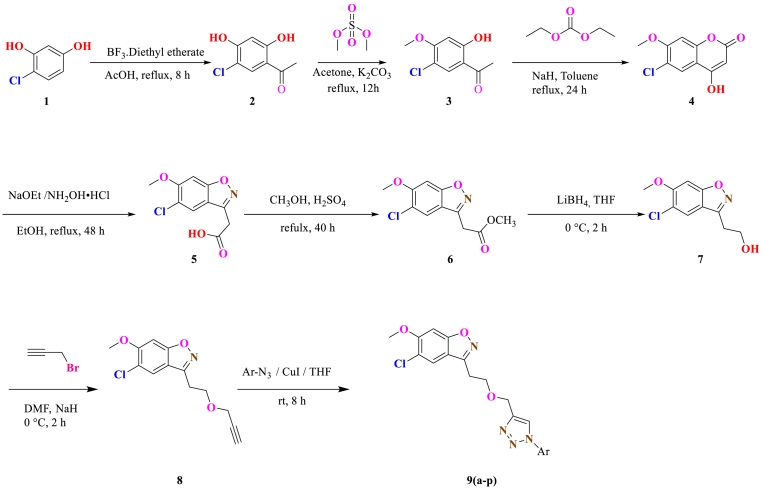
General synthetic route for the targeted compounds **9a-p**.

The synthetic approach adopted to obtain the 5-chloro-6-methoxy-3-(2-((1-(aryl)-1*H*-1,2,3-triazol-4-yl)methoxy)ethyl)benzo[*d*]isoxazole (**9a-o**) showed in Scheme 1. First, 4-chlorobenzene-1,3-diol (**1**) was reacted acetic acid in the presence of boron trifluoride diethyl etherate at 110 ^°^C for 12 h afforded 1-(5-chloro-2,4-dihydroxyphenyl)ethanone (**2**) followed by treatment with dimethyl sulfate in the presence of K_2_CO_3_ in acetone gave intermediate **3**. The compound 3 was cyclized with diethyl carbonate in the presence of NaH (60 % dispersion in mineral oil) yielede cyclized chromen-2-one product **4**. 2-(5-chloro-6-methoxybenzo[*d*]isoxazol-3-yl) acetic acid (**5**) was obtained by condensing compound **4** with hydroxylamine hydrochloride in the presence of sodium ethoxide followed by esterification to give **6** and reduction to yield **7** in a good yields. Next intermediate **7** was treated with propargyl bromide in DMF at 0 °C to get compound **8**. The Click reaction of compound **8** with various substituted aromatic azides yielded 1*H*-1,2,3- triazoles (**6a-l**) targeted 5-chloro-6-methoxy-3-(2-((1-(aryl)-1*H*-1,2,3-triazol-4-yl) methoxy) ethyl) benzo[*d*]isoxazole (**9a-o**) in a good yields. Here, different aryl azides, both electron-withdrawing groups (such as halide and CF_3_) and donating groups (such as methyl and methoxyl groups) are used for click chemistry reaction, 2-CF_3_ aryl azide reaction didn't work at 70 °C for 24 h; isolated 20 % yield. Both 2-CF_3_ and *O*-toluidine derivatives yielded low and other all substituted azide reactions provided in excellent yields.

### 2.4 Biological evolution

#### 2.4.1 SAR of α-glucosidase inhibitory assay

The ability of the synthesized derivatives to inhibit α-glucosidase was investigated and the results were listed in Table 3. All the final derivatives **9a-p** were displayed promising anti- α-glucosidase inhibitory activity with IC_50_ range 14.69–38.71 nmol compared to reference drug acarbose (IC_50_: 35.91 nmol). The results reveales that substituents at the R position affects on moderate to good activity of tested compounds. The unsubstituted phenyl derivative (9a, IC_50_ = 15.17 nmol) exhibited excellent inhibition, nearly two-fold more potent than the standard drug acarbose (IC_50_ = 35.91 nmol), suggesting that the benzo[*d*]isoxazole-triazole core contributes significantly to enzyme binding. Then introduction of electron-withdrawing group such as chlorine substitution at different positions had varying effects, with *ortho* (**9b**, IC_50_: 30.16 nmol) showing moderate activity, while meta (**9c**, IC_50_: 23.22 nmol), and *para* (**9d**, IC_50_: 23.22 nmol) positions of phenyl ring exhibited slightly better inhibition compared to **9b**. When R is substituted with *ortho*-fluro and *meta*-methyl containing group in phenyl ring such as **9e** (IC_50_: 30.16 nmol) showed slightly high inhibitory activity than acarbose (IC_50_: 35.91 nmol). The electron-donating group methyl (**9f**, IC_50_: 21.74 ± 4.52 μM) as R group displayeds a significant anti-*α*-glucosidase activity.

**Table 3:** Anti α-glucosidase inhibitory activity of hybrids **9a-o**.

Compound	Ar	IC_50_ (nmol)
**9a**	Ph	**15.17**
**9b**	2-ClC_6_H_4_	30.16
**9c**	3-ClC_6_H_4_	23.22
**9d**	4-ClC_6_H_4_	30.82
**9e**	2-F-3-H_3_C–C_6_H_3_	30.16
**9f**	2- H_3_CC_6_H_4_	21.74
**9g**	2-H_3_CO–C_6_H_4_	**18.68**
**9h**	4-H_3_CO–C_6_H_4_	38.71
**9i**	2-F-3-Br-C_6_H_3_	**16.19**
**9j**	4-FC_6_H_4_	**14.69**
**9k**	2-F_3_C–C_6_H_4_	**16.28**
**9l**	4-F_3_C–C_6_H_4_	28.71
**9m**	3.4-diMeC_6_H_3_	**16.19**
**9n**	4-BrC_6_H_4_	**14.69**
**9o**	3-F_3_C-4-Br-C_6_H_3_	**16.28**
**Acarbose**		**35.91**

For methoxy substitutions, the *ortho*-methoxy derivative (**9g**, IC_50_ = 18.68 nmol) demonstrated superior activity compared to *para*-methoxy substitution (**9h**, IC_50_ = 38.71 nmol), indicating that steric hindrance at the para position may reduce enzyme interaction. The bromine-substituted derivative (**9n**, IC_50_ = 14.69 nmol) exhibited a two-fold increase in activity compared to acarbose, suggesting that bulky halogens enhance binding interactions. Similarly, 3-CF_3_,4-Br substitution (**9o**, IC_50_ = 16.28 nmol) caused significant inhibition, likely due to the combined electron-withdrawing effects of CF_3_ and Br improving receptor binding. The influence of trifluoromethyl (-CF_3_) groups was position-dependent, with 2-CF_3_ (**9k**, IC_50_ = 35.91 nmol) slightly improving activity, while 4-CF_3_ (**9l**, IC_50_ = 28.71 nmol) led to reduced potency, indicating that positional effects impact enzyme interactions.

When R is replaced with 3,4-dimethyl substitution, **9m** exhibited the potential inhibition with IC_50_ of 28.71 nmol. Moreover, introduction of electron-withdrawing bromine group (**9n**) showed the two-fold increase in anti-α-glucosidase activity with IC_50_ of 14.69 nmol compared to standard drug acarbose (IC_50_: 35.91 nmol). Compound **9o** having 3-CF_3_,4-Br caused the significant inhibition of -glucosidase enzyme with IC_50_ of 16.28 nmol. The obtained results suggest that the hybridization pharmacophores such as benzo[*d*]isoxazole and triazole ring would be a promising strategy to develop a new potent α-glucosidase inhibitors as new anti-diabetic drug candidates.

#### 2.4.2 Antibacterial activity

Further, the antibacterial activity of the synthesized compounds was assessed against one Gram-negative *Escherichia coli* and one Gram-positive *Bacillus cereus* strains. Some of these compounds were exhibited strong zone of inhibition against tested strains and the results were displayed in Table 4. Against *Bacillus cereus* Compound **9f**, **9h**, and **9l** exhibited the strong zone of inhibition (0.5 mm) at concentration of 25 μl. Similarly, compound **9c** and **9l** showed 0.4 mm zone of inhibition against *Bacillus cereus.* Whereas compounds **9i**, **9j**, **9k**, **9m**, **9n** and **9o** exhibited the moderate inhibition against same strain at 25 μl concentration. Similarly, against *Escherichia coli,* only **9g** showed the strong zone of inhibition with value of 0.9 mm at 75 μl and 1.2 mm at 100 μl concentration. The rest other compounds didn't show any inhibition against *Escherichia coli* strain.

**Table 4:** Antibacterial activity of the compounds on *Bacillus cereus* and *Escherichia coli*.

Compound code	Zone of inhibition (mM)							
	Bacillus cereus				*Escherichia coli*			
	25 μl	50 μl	75 μl	100 μl	25 μl	50 μl	75 μl	100 μl
**9a**	0	0	0	0	0	0	0	0
**9b**	0	0	0	0	0	0	0	0
**9c**	0.4 ± 0.05	0.6 ± 0.12	0.4 ± 0.07	0.5 ± 0.02	0	0	0	0
**9d**	0	0	0	0	0	0	0	0
**9e**	0	0	0	0	0	0	0	0
**9f**	**0.5 ± 0.1**	**1.1 ± 0.15**	2 ± 0.2	2.2 ± 0.25	0	0	0	0
**9g**	0	0	1.1 ± 0.15	1.4 ± 0.1	0	0	**0.9 ± 0.05**	**1.2 ± 0.15**
**9h**	**0.5 ± 0.1**	**1.1 ± 0.15**	2 ± 0.2	2.2 ± 0.25	0	0	0	0
**9i**	0.2	0.3 ± 0.05	0.4 ± 0.1	0.4 ± 0.15	0	0	0	0
**9j**	0.2	0.2	0.3 ± 0.5	0.3 ± 0.5	0	0	0	0
**9k**	0.2	0.3 ± 0.05	0.3	0.4 ± 0.05	0	0	0	0
**9l**	**0.5 ± 0.1**	**1.1 ± 0.15**	2 ± 0.2	2.2 ± 0.25	0	0	0	0
**9m**	0.2	0.3 ± 0.05	0.4 ± 0.1	0.4 ± 0.15	0	0	0	0
**9n**	0.2	0.2	0.3 ± 0.5	0.3 ± 0.5	0	0	0	0
**9o**	0.2	0.3 ± 0.05	0.3	0.4 ± 0.05	0	0	0	0

mM: millimeter.

## 3 Conclusion

We have designed a series of novel 5-chloro-6-methoxy-3-(2-((1-(Aryl)-1*H*-1,2,3-triazol-4-yl)methoxy)ethyl)benzo[*d*]isoxazole derivatives by using molecular hybridization followed by molecular docking technique and synthesized *via* Cu(I) catalyzed click chemistry reaction in good yields. The highest docking score compounds were selected to synthesize and evaluate for their *in vitro α*-glucosidase inhibitory activity. To investigate the orientation, contact, and verification of the intended compounds on the *α*-glucosidase active site, docking studies were carried out. **9a** and **9j** had the highest docking energies with −9.2 and −9.4 kcal/mol, respectively. Compound **9a** showed strong hydrogen bonding with GLY-228 and GLU-271 and extensive hydrophobic interactions, while compound **9j** exhibited significant halogen bonding and π-interactions, emphasizing the role of electronic features in binding affinity. Further, all targeted compounds displayed strong *in vitro* α-glucosidase inhibitory activity with an IC_50_ range of 14.69–38.71 than control drug acarbose with IC_50_ value of 35.91 nmol. Notably, compounds 9a, 9g, 9n, and 9o showed superior inhibition, suggesting that both electronic and steric factors of the substituents at the R position play a pivotal role in modulating activity. Among them, **9j** and **9n** found to be the more promising α-glucosidase inhibitors (IC_50_: 14.69 nmol) followed by **9i** and **9m** (IC_50_: 16.19 nmol). The *in vitro* results were well supported to *in silico* results. In addition, compounds were subjected to investigate antibacterial effectiveness and found that **9f**, **9h**, and **9l** exhibited the strong zone of inhibition (0.5 mm) at concentration of 25 μl against *Bacillus cereus*. Additionally, the compounds were subjected to computational drug likeness/ADME testing, had good ADME and exhibited drug-like profiles. Collectively, the SAR and docking results confirm that the benzo[d]isoxazole-triazole scaffold, especially with suitable electron-withdrawing and hydrophobic substituents, holds promise as a lead framework for developing potent α-glucosidase inhibitors for managing type 2 diabetes.

## 4 Experimental section

### 4.1 Molecular docking

The docking studies are conducted using the PyRx-Virtual screening tool in conjunction with Auto Dock Vina [42]. Alpha-glucosidase's crystal structure (PDB ID:3WY1) was obtained from the RCSB PDB Database [43]. Using Swiss PDB Viewer, the protein was created by adding missing residues, eliminating other residues, and chain B [44].

The ligands were drawn using the Marvin sketch from Chem Axon, which has been optimized and clustered using the BIOVIA Discovery Studio Visualizer [45,46]. SD files were used to store the ligand files. Energy reduction was carried out after importing the clustered ligands into PyRx and creating PDBQT files,. After being loaded into PyRx, the protein was processed by eliminating water molecules, introducing hydrogens, and reducing energy.Based on the bound co-crystallized ligand with dimensions of center_x = −4.2, center_y = −20.4, center_z = 17.2, size_x = 25.0, size_y = 25.0, size_z = 27.7, grid properties were chosen. The PyRx virtual screening tool was used to bind the PDBQT files' ligands to the protein. For every ligand, we can obtain up to ten different positions; the best pose is then chosen. Using PyMOL and BIOVIA Discovery Studio Visualizer, the optimal docked ligand conformations, bond lengths, bond angles, and bonding interactions were examined.

### 4.2 Evaluation of physico-chemical properties

The compounds' SMILES representations and corresponding chemical codes were input into SwissADME [47], a freely accessible web tool (http://www.swissadme.ch/index.php). The application has a "run" button function that helps with parameter computation after the submission process is complete. The collected data was then subjected to data analysis after being made available in both PDF and CSV formats.

### 4.3 Chemicals and instruments

Without additional purification, all solvents and reagents were utilized after being bought from Sigma Aldrich Chemicals Limited in India. Melting points were measured in open capillaries without correction using the Stuart SMP30 instrument. Using F254 silica-gel precoated sheets and hexane/ethyl acetate (7.5/2.5) as the eluent, thin layer chromatography was used to assess the reactions' progress and the products' purity. ^1^H NMR and ^13^C NMR spectra were recorded on Bruker spectrometer at 400 MHz, CDCl_3_ and DMSO-d_6_ as the solvent and TMS as the internal standard. Mass spectral measurements were performed at 70 eV using the multimode approach (EI and APCI) on a Shimadzu spectrometer.

### 4.4 General synthetic route

#### 4.4.1 Synthesis of 1-(5-Chloro-2,4-dihydroxyphenyl)ethanone (2)

To a stirred solution of 4-chlorobenzene-1,3-diol (**1**) (20 g, 138.3 mmol) in acetic acid (100 mL) was added boron trifluoride diethyletherate (276.6 mL, 276.6 mmol) under Ar atmosphere and stirred the reaction mixture at 100 ^°^C for 12 h. After completion of the reaction by TLC analysis, the reaction mixture was poured into ice-cold water (500 mL) and stirred for 1 h. After filtration, the obtained solid was dried in an oven for 4 h at 80 ^°^C to obtain the crude product. This was re-crystallized with diethyl ether (150 mL) to afford 1-(5-chloro-2,4-dihydroxyphenyl) ethanone **(2)** (23 g, 89 % yield) as a light brown solid. M.P: 151–153 °C: ^1^H NMR (400 MHz, DMSO-*d*_*6*_): *δ* 12.35 (s, 1H), 11.40 (s, 1H), 7.84 (s, 1H), 6.40 (s, 1H), 2.58 (s, 3H); MS (EI): 184.93 [M]^-^. TLC (R_f_ 0.4 in 1:1 EtOAc/hexane)

#### 4.4.2 Synthesis of 1-(5-Chloro-2-hydroxy-4-methoxyphenyl)ethanone (3)

To a mixture of 1-(5-chloro-2,4-dihydroxyphenyl)ethanone (**2**) (22 g, 117.9 mmol) in acetone (300 mL) were added K_2_CO_3_ (48.9 g, 353.7 mmol) and dimethyl sulfate (29.7 g, 235.8 mmol) at rt and stirred at 56 °C for 12 h. Upon completion of compound **2** by thin layer chromatography (TLC), the reaction product was filtered through a celite pad, and the filtrate was evaporated under decreased pressure to afford crude compound. The product was re-crystallized with ethanol (100 mL) to afford of 1-(5-chloro-2-hydroxy-4-methoxy phenyl) ethanone (**3**) (20 g, 84 % yield) as an off-white solid. M.P: 138–140 °C: ^1^H NMR (400 MHz, DMSO-*d*_*6*_): *δ* 12.59 (s, 1H), 7.94 (s, 1H), 6.69 (s, 1H), 3.91(S, 3H) 2.58 (s, 3H); MS (EI): 199.06 [M]^-^. TLC (R_f_ 0.5 in 1:1 EtOAc/hexane)

#### 4.4.3 Synthesis of 6-Chloro-4-hydroxy-7-methoxy-2*H*-chromen-2-one (4)

To a stirred suspension of 60 % NaH (3.42g, 142.5 mmol) in dry toluene (200 mL) was added 1-(5-chloro-2-hydroxy-4-methoxyphenyl)ethanone (19 g, 99 mmol) and diethyl carbonate (15.2 g, 128.7 mmol) under Ar atmosphere at rt and stirred at 110 °C for 12 h. After completion of the compound-**3** by TLC analysis, the reaction mixture was poured into ice-cold water (200 mL) and stirred for 1 h. The obtained solid was filtered and dried to give a crude product. The product was re-crystallized with ethanol (150 mL) to afford 6-chloro-4-hydroxy-7-methoxy-2*H*-chromen-2-one (**4**) (18.0 g 88 % yield) as an off-white solid. MP: >300 °C. ^1^H NMR (400 MHz, DMSO-*d*6): *δ* 12.58 (bs, 1H), 7.76 (s, 1H), 7.20 (s, 1H), 5.49 (s, 1H), 3.95 (s, 3H); MS (EI): 227.0 [M]. TLC (R_f_ 0.3 in 1:1 EtOAc/hexane)

#### 4.4.4 Synthesis of 2-(5-Chloro-6-methoxybenzo[*d*]isoxazol-3-yl)acetic acid (5)

A mixture of hydroxylamine hydrochloride (13.6 g, 413.5 mmol) and sodium ethoxide (28.1 g, 413.5 mmol) in ethanol (200 mL) was treated with 6-chloro-4-hydroxy-7-methoxy-2*H*-chromen-2-one (**4)** (20 g, 82.7 mmol) at 90 ^°^C for 48 h. After completion of the reaction by TLC analysis, the reaction mixture was cooled to 0 °C, poured into 1 N HCl solution (200 mL), and stirred for 4 h. The obtained solid was filtered and washed with water (150 mL) and dried under a vacuum to give a crude compound. The product was recrystallized with ethanol (150 mL) to afford 2-(5-chloro-6-methoxybenzo[*d*]isoxazol-3-yl)acetic acid (**5**) (16.3 g, 76 % yield) as an off-white solid. MP: 158–160 °C: ^1^H NMR (400 MHz, DMSO-*d*_6_): *δ* 12.59 (bs, 1H), 7.96 (s, 1H), 7.52 (s, 1H), 4.18 (s, 2H), 3.96 (s, 3H); MS (EI): 242.0 [M]. TLC (R_f_ 0.2 in 10 % CH_3_OH/CH_2_Cl_2_).

#### 4.4.5 Methyl 2-(5-chloro-6-methoxybenzo[*d*]isoxazol-3-yl)acetate (6)

To a stirred solution of 2-(5-chloro-6-methoxybenzo[*d*]isoxazol-3-yl)acetic acid (**5**) (7.0 g, 20.7 mmol) in anhydrous methanol (200 mL) was added sulfuric acid (10 mL) and stirred at reflux for 40 h. After completion of the reaction by TLC analysis, excess methanol was removed under reduced pressure. The reaction mixture was neutralized with sat. sodi bicarbonate solution (100 mL) and extracted with EtOAc (2 × 150 mL), the combined organic layer was washed with brine (100 mL), dried over anhydrous sodium sulfate, filtered, and concentrated under reduced pressure to afford crude compound. The crude compound was purified by column chromatography using silica (100–200 mesh) eluting with 50 % EtOAc in hexanes to afford methyl 2-(5-chloro-6-methoxybenzo[*d*]isoxazol-3-yl)acetate (6) (5.5 g, 74 % yield) as an off-white solid. MP: 160–165 °C: ^1^H NMR (400 MHz, DMSO-*d*_*6*_): *δ* 7.99 (s, 1H), 7.55 (s, 1H), 4.18 (s, 2H), 3.97 (s, 3H), 3.68 (s, 3H), MS (ES + APCI): 256.0 [M+H]^+^. TLC (R_f_ 0.4 in 7:3 EtOAc/hexane).

#### 4.4.6 Synthesis of 2-(5-Chloro-6-methoxybenzo[*d*]isoxazol-3-yl)ethanol (7)

To a stirred solution of methyl 2-(5-chloro-6-methoxybenzo[*d*]isoxazol-3-yl)acetate **(6)** (5 g, 19.5 mmol) in anhydrous THF was added lithium borohydride (2.0 M in THF) (29.2 mL, 58.5 mmol) dropwise over a period of 5 min at 0 ^°^C. The reaction mixture was slowly allowed to rt and stirred for 2 h. After completion of the reaction by TLC analysis, the reaction mixture was quenched with sat. ammonium chloride solution (50 mL) at 0 °C and extracted with EtOAc (2 × 100 mL). The organic layer was washed with brine (100 mL), dried over anhydrous sodium sulfate, and concentrated under reduced pressure to afford crude compound. The product was purified by column chromatography using silica 100–200 mesh eluted with 70 % EtOAc in hexanes to afford 2-(5-chloro-6-methoxybenzo[*d*]isoxazol-3-yl)ethanol (**7**) (3.5 g, 79 %) as an off-white solid.

M.P: 98–100 °C: ^1^H NMR (400 MHz, DMSO-*d*_*6*_): *δ* 8.02 (s, 1H), 7.49 (s, 1H), 4.91 (t, *J* = 4.0 Hz, 1H), 3.96 (s, 3H), 3.81 (q, *J* = 4.0 Hz, 2H), 3.07 (t, *J* = 4.0 Hz, 2H), MS (EI): 228.0 [M+H]. TLC (R_f_ 0.3 in 7:3 EtOAc/hexane)

#### 4.4.7 Synthesis of 5-Chloro-6-methoxy-3-(2-(prop-2-yn-1-yloxy)ethyl)benzo[*d*]isoxazole (8)

To a stirred suspension of 60 % NaH (1.22 g, 30.6 mmol) in dry DMF (200 mL) was added 2-(5-chloro-6-methoxybenzo[*d*]isoxazol-3-yl)ethanol (**7**) (3.50 g, 15.3 mmol) under Ar atmosphere at 0 °C and stirred for 30 min. Propargyl bromide (2.73 g, 22.9 mmol) was added dropwise and stirred for 2 h at 0 °C. The reaction mixture was monitored by TLC. Upon completion of compound 7, the reaction mixture was poured into ice-cold water (100 mL) and extracted with EtOAc (2 × 100 mL). The combined organic layer was washed with brine solution (100 mL) and dried over sodium sulfate, filtered, and concentrated under reduced pressure to give a crude product. The product was purified by column chromatography eluting with 50 % EtOAc in hexanes to afford 5-chloro-6-methoxy-3-(2-(prop-2-yn-1-yloxy)ethyl)benzo[*d*]isoxazole (**8**) (2.5 g, 95 % yield) as an off white solid. MP: 55–58 °C: ^1^H NMR (400 MHz, DMSO-*d*_6_): *δ* 8.03 (s, 1H), 7.50 (s, 1H), 4.16 (d, *J* = 2.4 Hz, 1H), 3.96 (s, 3H), 3.85 (t = 6.4 Hz, 2H), 3.42 (t, *J* = 2.0 Hz, 1H), 3.19 (t, *J* = 6.4 Hz, 2H): MS (EI + APCI): 266 [M+H]^+^. TLC (R_f_ 0.4 in 1:1 EtOAc/hexane)

#### 4.4.8 General procedure for the synthesis of 5-chloro-6-methoxy-3-(2-((1-phenyl-1*H*-1,2,3-triazol-4-yl)methoxy)ethyl)benzo[*d*]isoxazole (9a-p)

To a solution of 5-chloro-6-methoxy-3-(2-(prop-2-yn-1-yloxy)ethyl)benzo[*d*]isoxazole **(8)** (200 mg, 0.754 mmol) in anhydrous THF (5.0 mL) were added aryl azide (1.13 mmol) and CuI (0.114 mmol) under Ar atmosphere and stirred at rt for 24 h. After completion of the reaction by TLC analysis (R_f_ 0.4 to 0.5 for all derivative in 7:3 EtOAc/hexanes), the reaction mixture was filtered through a celite pad and washed with EtOAc (20 mL), the filtrate was concentrated under reduced pressure to afford crude compound. The product was purified by column chromatography (silica 100–200 mesh) eluting with 30 %–50 % EtOAc in hexanes pure fractions were combined and concentrated under reduced pressure to afford 5-chloro-6-methoxy-3-(2-((1-aryl-1*H*-1,2,3-triazol-4-yl)methoxy)ethyl)benzo[*d*]isoxazole (**9a-o**) in good to excellent yields.

5-Chloro-6-methoxy-3-(2-((1-phenyl-1*H*-1,2,3-triazol-4-yl)methoxy)ethyl)benzo[*d*] isoxazole (9a)

Off-white solid. Yield 54 %. MP: 78–80 °C, IR (KBr): υ (cm^−1^) 3294, 3174, 2980, 2859, 2711, 2047, 1738, 1515, 1355, 875. ^1^H NMR (400 MHz, CDCl_3_): *δ* 7.88 (s, 1H), 7.72 (s, 1H), 7.71–7.69 (m, 2H), 7.54–7.50 (m, 2H), 7.46–7.42 (m, 1H), 7.02 (s, 1H), 4.75 (s, 2H), 3.98 (t, *J* = 6.4 Hz, 2H), 3.95 (s, 3H), 3.23 (t, *J* = 6.4 Hz, 2H). ^13^C NMR (100 Mz, CDCl_3_): *δ*163.1, 156.9, 156.1, 145.5, 136.9, 129.7 (3C, Ar), 128.7, 122.3, 120.6 (2C, Ar), 119.8, 115.2, 92.6, 68.2, 64.6, 56.5, 26.2. MS (ES + APCI): 385.1 [M+H]^+^. TLC (R_f_ 0.4 in 7:3 EtOAc/hexane)

5-Chloro-3-(2-((1-(2-chlorophenyl)-1*H*-1,2,3-triazol-4-yl)methoxy)ethyl)-6-methoxybenzo[*d*]isoxazole (9b)

Off-white solid. Yield 73 %. MP: 80–82 °C, IR (KBr): υ (cm^−1^) 3237, 3180,2897, 2854, 2716, 2219, 1744, 1607, 1326, 867. ^1^H NMR (400 MHz, CDCl_3_): *δ* 7.89 (s, 1H), 7.71 (s, 1H), 7.62–7.56 (m, 2H), 7.47–7.44 (m, 2H), 7.02 (s, 1H), 4.78 (s, 2H), 3.99 (t, *J* = 6.4 Hz, 2H), 3.96 (s, 3H), 3.23 (t, *J* = 6.4 Hz, 2H). ^13^C NMR (100 Mz, CDCl_3_): *δ* 163.1, 156.9, 156.0, 144.5, 134.8, 130.7 (2C, Ar), 128.5, 127.9, 127.8, 124.6, 122.3, 119.8, 115.2, 92.6, 68.2, 64.5, 56.5, 26.2. MS (ES + APCI): 419 [M+H]^+^. TLC (R_f_ 0.4 in 7:3 EtOAc/hexane)

5-Chloro-3-(2-((1-(3-chlorophenyl)-1*H*-1,2,3-triazol-4-yl)methoxy)ethyl)-6-methoxybenzo[*d*]isoxazole (9c)

Off-white solid. Yield 75 %. MP: 86–88 °C, IR (KBr): υ (cm^−1^) 3197, 3150, 2985, 2808, 2711, 2642, 1761, 1572, 1378, 869. ^1^H NMR (400 MHz, CDCl_3_): *δ* 7.86 (s, 1H), 7.71 (t, *J* = 1.96 Hz, 1H), 7.70 (s, 1H), 7.65–7.59 (m, 1H), 7.50–7.40 (m, 2H), 7.02 (s, 1H), 4.75 (s, 2H), 3.98 (t, *J* = 6.4 Hz, 2H), 3.96 (s, 3H), 3.23 (t, *J* = 6.4 Hz, 2H). ^13^C NMR (100 Mz, CDCl_3_): *δ* 163.1, 156.9, 156.0, 144.5, 134.8, 130.7 (2C, Ar), 128.5, 127.9, 127.8, 124.6, 122.3, 119.8, 115.2, 92.6, 68.2, 64.5, 56.5, 26.2. MS (ES + APCI): 419 [M+H]^+^. TLC (R_f_ 0.4 in 7:3 EtOAc/hexane)

5-Chloro-3-(2-((1-(4-chlorophenyl)-1*H*-1,2,3-triazol-4-yl)methoxy)ethyl)-6-methoxybenzo[*d*]isoxazole (9d)

Off-white solid. Yield 80 %. MP: 85–87 °C, IR (KBr): υ (cm^−1^) 3391, 3191, 2894, 2814, 2705, 2247, 1784, 1710, 1647, 1424, 903. ^1^H NMR (400 MHz, CDCl_3_): *δ* 7.84 (s, 1H), 7.69–7.65 (m, 3H), 7.51–7.48 (m, 2H), 7.01 (s, 1H), 4.74 (s, 2H), 3.98 (t, *J* = 6.4 Hz, 2H), 3.96 (s, 3H), 3.22 (t, *J* = 6.4 Hz, 2H). ^13^C NMR (100 Mz, CDCl_3_): *δ* 163.1, 157.0, 156.1, 145.8, 135.4, 134.5, 129.2 (2C, Ar), 122.3, 121.7 (2C, Ar), 120.5, 119.7, 115.2, 92.6, 68.2, 64.6, 56.5, 26.2. MS (ES + APCI): 419 [M+H]^+^. TLC (R_f_ 0.4 in 7:3 EtOAc/hexane)

5-Chloro-3-(2-((1-(2-fluoro-3-methylphenyl)-1*H*-1,2,3-triazol-4-yl)methoxy)ethyl)-6-methoxybenzo[*d*]isoxazole (9e)

Off-white solid. Yield 80 %. M.P: 100–105 °C, IR (KBr): υ (cm^−1^) 3191, 3151, 2928, 2802, 2716, 2041, 1767, 1527, 1372, 875. ^1^H NMR (400 MHz, DMSO-d_*6*_): *δ* 8.46 (d, *J* = 2.0 Hz, 1H), 7.99 (s, 1H), 7.60 (t, *J* = 7.70 Hz, 1H), 7.81–7.47 (m, 2H), 7.32 (t, *J* = 7.70 Hz, 1H), 4.66 (s, 2H), 3.96 (s, 3H), 3.92 (t, *J* = 6.4 Hz, 2H), 3.22 (t, *J* = 6.4 Hz, 2H), 2.35 (d, *J* = 1.6 Hz, 3H). ^13^C NMR (100 Mz, DMSO-d_*6*_): *δ* 161.9, 155.9, 155.5, 143.7, 131.7, 125.8, 125.6, 124.7, 124.1, 124.0, 122.7, 121.8, 118.0, 114.0, 91.7, 66.5, 62.4, 56.2, 24.7, 13.5. MS (ES + APCI): 417.1 [M+H]^+^. TLC (R_f_ 0.5 in 7:3 EtOAc/hexane)

##### 4.4.8.1 5-Chloro-6-methoxy-3-(2-((1-(*o*-tolyl)-1*H*-1,2,3-triazol-4-yl)methoxy)ethyl)benzo[*d*] isoxazole (9f)

Off-white solid. Yield 33 %. MP: 50–55 °C: IR (KBr): υ (cm^−1^) 3191, 3174, 2934, 2796, 2722, 2624, 1750, 1532, 1378, 880. ^1^H NMR (400 MHz, CDCl_3_): *δ* 7.72 (s, 1H), 7.64 (s, 1H), 7.44–7.29 (m, 4H), 7.02 (s, 1H), 4.73 (s, 2H), 3.99 (t, *J* = 6.6 Hz, 2H), 3.96 (s, 3H), 3.22 (t, *J* = 6.6 Hz, 2H), 2.19 (s, 3H). ^13^C NMR (100 Mz, CDCl_3_): *δ* 163.1, 156.9, 156.1, 144.6, 136.4, 133.6, 131.4, 129.8, 126.8, 125.9, 124.0, 122.3, 119.8, 115.2, 92.6, 68.2, 64.6, 56.5, 26.2, 17.8. MS (ES + APCI): 399.1 [M+H]^+^. TLC (R_f_ 0.5 in 7:3 EtOAc/hexane)

5-Chloro-6-methoxy-3-(2-((1-(2-methoxyphenyl)-1*H*-1,2,3-triazol-4-yl)methoxy)ethyl) benzo[*d*]isoxazole (9g)

Off-white solid. Yield 64 %. MP: 70–72 °C: IR (KBr): υ (cm^−1^) 3185, 3174, 2928, 2805, 2785, 2716, 1664, 1567, 1155, 1029, 680. ^1^H NMR (400 MHz, CDCl_3_): *δ* 8.60 (s, 1H), 7.98 (s, 1H), 7.81–7.68 (m, 2H), 7.47 (s, 1H), 7.14–7.11 (m, 2H), 4.63 (s, 2H), 3.99–3.87(m, 5H), 3.83 (s, 3H), 3.21 (t, *J* = 6.2 Hz, 2H). ^13^C NMR (100 Mz, CDCl_3_): *δ* 163.0, 159.6 156.9, 156.6, 145.1, 130.5, 130.7 (2C, Ar), 122.9, 122.5, 122.1, 119.0, 115.2, 115.1, 93.6, 67.5, 63.7, 57.3, 56.0, 25.8. (ES + APCI): 415 [M+H]^+^. TLC (R_f_ 0.4 in 7:3 EtOAc/hexane)

5-Chloro-6-methoxy-3-(2-((1-(4-methoxyphenyl)-1*H*-1,2,3-triazol-4-yl)methoxy)ethyl) benzo[*d*]isoxazole (9h)

Off-white solid. Yield 68 %., MP: 80–85 °C; IR (KBr): υ (cm^−1^) 3237, 3128, 2945, 2968, 1635, 1561, 1418, 1378, 1041, 806. ^1^H NMR (400 MHz, CDCl_3_): *δ* 7.79 (s, 1H), 7.71 (s, 1H), 7.67–7.54 (m, 2H), 7.05–6.97 (m, 3H), 4.74 (s, 2H), 3.98 (t, *J* = 6.1 Hz, 2H), 3.96 (s, 3H), 3.87 (s, 3H), 3.22 (t, *J* = 6.1 Hz, 2H). ^13^C NMR (100 Mz, CDCl_3_): *δ* 163.1, 159.8, 156.9, 156.1, 145.3, 130.4, 122.4, 122.2 , 120.8, 119.7, 115.2, 114.7, 114.3, 92.6, 68.1, 64.6, 56.5, 56.6, 26.2, 26.0. MS (ES + APCI): 415 [M+H]^+^. TLC (R_f_ 0.4 in 7:3 EtOAc/hexane)

3-(2-((1-(4-Bromo-3-fluorophenyl)-1*H*-1,2,3-triazol-4-yl)methoxy)ethyl)-5-chloro-6-methoxybenzo[*d*]isoxazole (9i)

Off-white solid. Yield 63 %. M.P: 90–95 °C: IR (KBr): υ (cm^−1^) 3437, 3185, 2939, 2911, 2699, 1784, 1578, 1361, 886. ^1^H NMR (400 MHz, DMSO-d_*6*_): *δ* 8.80 (s, 1H), 8.06–7.89 (m, 3H), 7.89–7.69 (m, 1H), 7.48 (s, 1H), 4.64 (s, 2H), 4.00–3.85 (m, 5H), 3.20 (t, *J* = 6.3 Hz, 2H). ^13^C NMR (100 MHz, DMSO-d_*6*_): 163.0, 157.7, 156.9, 156.5, 145.6, 137.5, 135.1, 122.5, 122.8,119, 117.6, 115.0, 109.2, 108.9, 93.9, 67.4, 63.5, 57.3, 25.8. MS (ES + APCI): 481 [M+H]^+^. TLC (R_f_ 0.5 in 7:3 EtOAc/hexane)

5-Chloro-3-(2-((1-(4-fluorophenyl)-1*H*-1,2,3-triazol-4-yl)methoxy)ethyl)-6-methoxy benzo[*d*]isoxazole (9j)

Off-white solid. Yield 60 %., M.P: 110–115 °C: ]^+^. IR (KBr): υ (cm^−1^) 3277, 3163, 2974, 2945, 1653, 1630, 1464, 1326, 1218, 898. ^1^H NMR (400 MHz, DMSO-d_*6*_): *δ* 8.71 (s, 1H), 7.97 (s, 1H), 7.95–7.84 (m, 2H), 7.56–7.29 (m, 3H), 4.64 (s, 2H), 4.01–3.82 (m, 5H), 3.21 (t, *J* = 6.4 Hz, 2H). ^13^C NMR (100 MHz, DMSO-d_*6*_): *δ* 163.0, 160.8, 156.9, 156.6, 145.3, 133.6, 122.9, 122.8 (2C, Ar), 122.7, 119.0, 117.2, 117.0, 115.0, 93.9, 67.5, 63.6, 57.3, 25.8. MS (ES + APCI): 403 [M+H]^+^. TLC (R_f_ 0.5 in 7:3 EtOAc/hexane)

5-Chloro-6-methoxy-3-(2-((1-(2-(trifluoromethyl)phenyl)-1*H*-1,2,3-triazol-4-yl)methoxy)ethyl)benzo[*d*]isoxazole (9k)

Off-white solid. Yield 20 %. MP: 100–105 °C, IR (KBr): υ (cm^−1^) 3180, 3157, 2980, 2991, 1618, 1607, 1595, 1447, 1321, 1275, 949., ^1^H NMR (400 MHz, CDCl_3_): *δ* 7.86 (dd, *J* = 7.56, 1.16 Hz,1H), 7.80–7.66 (m, 4H), 7.60–7.53 (m, 1H), 7.03 (s, 1H), 4.78 (s, 2H), 4.07–3.88 (m, 5H), 3.22 (t, *J* = 6.2 Hz, 2H). MS (ES + APCI): 453 [M+H]^+^. TLC (R_f_ 0.4 in 7:3 EtOAc/hexane)

5-chloro-6-methoxy-3-(2-((1-(4-(trifluoromethyl)phenyl)-1*H*-1,2,3-triazol-4-yl)methoxy)ethyl)benzo[*d*]isoxazole (9l)

Off-white solid. Yield 60 %, M.P: 90–95 °C, IR (KBr): υ (cm^−1^) 3185, 3105, 2951, 2928, 2774, 1773, 1515, 1338, 863., ^1^H NMR (400 MHz, DMSO-d_*6*_): *δ* 8.88 (s, 1H), 8.23–8.09 (m, 2H), 8.03–7.89 (m, 3H), 7.45 (s, 1H), 4.66 (s, 2H), 4.03–3.95 (m, 5H), 3.22 (t, *J* = 6.3 Hz, 2H). ^13^C NMR (100 MHz, DMSO-d_*6*_): *δ* 163.0, 156.9 (2C, Ar), 156.5 (2C, Ar), 145.7, 139.8, 127.6, 127.5, 125.7, 122.9, 122.8, 120.8, 119.0, 115.0, 93.9, 67.5, 63.5, 57.3, 25.8. (ES + APCI): 453 [M+H]^+^. TLC (R_f_ 0.4 in 7:3 EtOAc/hexane)

5-Chloro-3-(2-((1-(3,4-dimethylphenyl)-1*H-*1,2,3-triazol-4-yl)methoxy)ethyl)-6-methoxy benzo[*d*]isoxazole (9 m)

Off-white solid. Yield 57 %, M.P: 70–75 °C, IR (KBr): υ (cm^−1^) 3506, 3265, 3134, 2905, 2894, 2728, 1681, 1595, 1544, 989, 963. ^1^H NMR (400 MHz, DMSO-d_*6*_): *δ* 8.63 (s, 1H), 7.98 (s, 1H), 7.66 (d, *J* = 1.96 Hz, 1H), 7.55 (dd, *J* = 8.0, 2.2 Hz, 1H), 7.46 (s, 1H), 7.32 (d, *J* = 8.21 Hz, 1H), 4.63 (s, 2H), 4.00–3.83 (m, 5H), 3.21 (t, *J* = 6.3 Hz, 2H), 2.31 (s, 3H), 2.28 (s, 3H). ^13^C NMR (100 MHz, DMSO-d_*6*_): *δ* 163.0, 156.9, 156.6, 145.1, 138.5, 137.3, 134.9, 130.9, 122.9, 122.3, 121.2, 119.0, 117.6, 115.1, 93.9, 67.5, 63.7, 57.3, 25.8, 19.8, 19.4. MS (ES + APCI): 413 [M+H]+. TLC (R_f_ 0.3 in 7:3 EtOAc/hexane)

3-(2-((1-(4-Bromophenyl)-1*H*-1,2,3-triazol-4-yl)methoxy)ethyl)-5-chloro-6-methoxy benzo[*d*]isoxazole (9n)

Off-white solid. Yield 73 %. MP: 100–105 °C, IR (KBr): υ (cm^−1^) 3174, 3100, 2957, 2911, 2756, 1767, 1527, 1355, 892. ^1^H NMR (400 MHz, CDCl_3_): *δ* 7.84 (s, 1H), 7.69 (s, 1H), 7.68–7.59 (m, 4H), 7.01 (s, 1H), 4.74 (s, 2H), 3.98 (t, *J* = 6.2 Hz, 2H), 3.96 (s, 3H), 3.22 (t, *J* = 6.2 Hz, 2H). ^13^C NMR (100 MHz, CDCl_3_): *δ* 163.1, 156.9, 156.1, 145.9, 135.9, 132.8 (2C, Ar), 122.4, 122.3, 121.9 (2C, Ar), 120.4, 119.7, 115.2, 92.6, 68.2, 64.5, 56.5, 26.2. MS (ES + APCI): 463 [M+H]+. TLC (R_f_ 0.5 in 7:3 EtOAc/hexane)

3-(2-((1-(4-Bromo-3-(trifluoromethyl)phenyl)-1*H*-1,2,3-triazol-4-yl)methoxy)ethyl)-5-chloro-6-methoxybenzo[*d*]isoxazole (9o)

Off-white solid. Yield 80 %, M.P: 110–115 °C: IR (KBr): υ (cm^−1^) 3128, 3105, 2957, 2917, 2762, 1790, 15110, 1527, 1326, 817. ^1^H NMR (400 MHz, DMSO-d_6_): *δ* 8.93 (s, 1H), 8.31 (s, 1H), 8.13–8.12 (m, 2H), 7.94 (s, 1H), 7.45 (s, 1H), 4.65 (s, 2H), 3.93 (t, *J* = 6.3 Hz, 2H), 3.91 (s, 3H), 3.20 (t, *J* = 6.3 Hz, 2H). ^13^C NMR (100 MHz, DMSO-d_6_): *δ* 163.0, 156.9, 156.5, 145.7, 137.1, 136.4, 130.3, 130, 125.5, 123.0, 121.5, 119.8, 118.9, 118.5, 115.0, 93.9, 67.4, 63.5, 57.3, 25.8. MS (ES + APCI): 531 [M+H]+. TLC (R_f_ 0.5 in 7:3 EtOAc/hexane)

### 4.5 α-Glucosidase inhibitory assay method

The method outlined by Pistia Brueggeman et al. [48] and Hollingsworth et al. [49] was utilized to evaluate the inhibitory activity of α-glucosidase, with some modifications. Different doses ranging from 12.5 to 400 μg/mL were incubated with plant extracts in quantities of 50 μL. A 20-min incubation period was conducted at 37 °C using 10 μL of an enzyme solution (1 U/mL) containing α-glucosidase (maltase). A further addition of 125 μL of pH 6.8 % 0.1 M phosphate buffer was then added. The mixture was left to incubate for 30 min after 20 min, during which time 20 μL of 1 M pNPG (substrate) was added to start the reaction. A final absorbance measurement was made at 405 nm after 0.1 N of Na_2_CO_3_ (50 μL) was added to stop the reaction. As a positive control, acarbose was used at dosages ranging from 12.5 to 400 μg/mL. Enzyme activity was calculated as: (ODBLANK-ODSAMPLE)/ODBLANK x 100.

The amount of α-glucosidase enzyme required to generate 1 μmol of the product (*p*-nitrophenol) from the substrate (*p*-nitrophenyl-α-d-glucopyranoside) in a minute is a precise representation of one unit of the enzyme. By fitting a regression equation to a plot of concentration (ranging from 12.5 to 400 μg/mL) on the x-axis and % inhibition on the y-axis for different extracts and fractions, the IC_50_, or the concentration required to inhibit 50 % of the enzyme activity was found.

### 4.6 Antimicrobial activity

The antibacterial activity was determined by using the agar well diffusion method [[50], [51], [52]]. Two bacterial strains were used to evaluate the antibacterial efficacy of the synthesized compounds: one Gram-positive strain, *Bacillus cereus* (MTCC2128), and one Gram-negative strain, *Escherichia coli* (MTCC2412). Sterilized Mueller Hinton Broth (MHB) was used to cultivate the bacterial strains, and the mixture was incubated for 18 h at 37 °C. To evaluate the antibacterial activity, the inhibition zone (RIZ) radius encircled each well was determined in millimeters. The experiments were carried out duplicates. The incubation period for the plates was from 72 to 96 h at a temperature of 37 °C. The zone of inhibition was then looked for on the plates.

## CRediT authorship contribution statement

**Ram Reddy Mudireddy:** Writing – original draft, Methodology, Investigation, Formal analysis. **Rambabu Gundla:** Supervision, Project administration. **Chandra Prakash Koraboina:** Visualization, Conceptualization. **Vani Madhuri Velavalapalli:** Software. **Venkata Veernjaneya Sarma Dhulipalla:** Validation, Resources. **Gowri Sankararao Burle:** Funding acquisition, Data curation. **Sreekantha B. Jonnalagadda:** Writing – review & editing, Funding acquisition. **Naresh Kumar Katari:** Writing – review & editing.

## Ethics approval

This article does not contain any studies with animals performed by any of the authors.

## Consent for publication

We authorize to publish the article without any conflict.

## Declaration of competing interest

The authors declare that no conflict of interest to publish this article.

## Data Availability

No data was used for the research described in the article.
