# Supplementary material for: Novel and efficient synthesis of 5-chloro-6-methoxy-3-(2-((1-(aryl)-1H-1,2,3-triazol-4-yl)methoxy)ethyl)benzo[d]isoxazole derivatives as new α-glucosidase inhibitors
Source: Biochem Biophys Rep. 2025 Jun 5;43:102074. doi: 10.1016/j.bbrep.2025.102074 (PMC12179616; doi:10.1016/j.bbrep.2025.102074)
Supplement: Multimedia component 1 [file mmc1.docx]

**Novel and Efficient Synthesis of 5-Chloro-6-methoxy-3-(2-((1-(aryl)-1*H*-1,2,3-triazol-4-yl)methoxy)ethyl)benzo[*d*]isoxazole Derivatives as New *α*-Glucosidase Inhibitors**

Ram Reddy Mudireddy^1,2^, Rambabu Gundla^*,1^, Chandra Prakash Koraboina^1^, Vani Madhuri Velavalapalli^1^, Venkata Veernjaneya Sarma Dhulipalla^3^, Gowri Sankararao Burle^4^, Sreekantha B Jonnalagadda^5^, Naresh Kumar Katari^5*^

^1^Department of Chemistry, School of Science, GITAM (Deemed to be University) Hyderabad, Telangana - 502 329, India.

^2^B.V.Raju Institute of Technology, Vishnupur, Narsapur, Medak Dist, Telangana - 502313, India.

^3^Analytical R&D, Catalent pharma solutions, 2725 Scherer Drive, St. Petersburg, FL 33716

^4^Research and Development, HIKMA Pharmaceuticals, 1809 N Wilson Rd, Columbus, OH 43228, USA

^5^School of Chemistry & Physics, College of Agriculture, Engineering & Science, Westville Campus, University of KwaZulu-Natal, P Bag X 54001, Durban-4000, South Africa.

**Correspondence Email:** [rgundla@gitam.edu](mailto:rgundla@gitam.edu); [KatariN@ukzn.ac.za](mailto:KatariN@ukzn.ac.za)


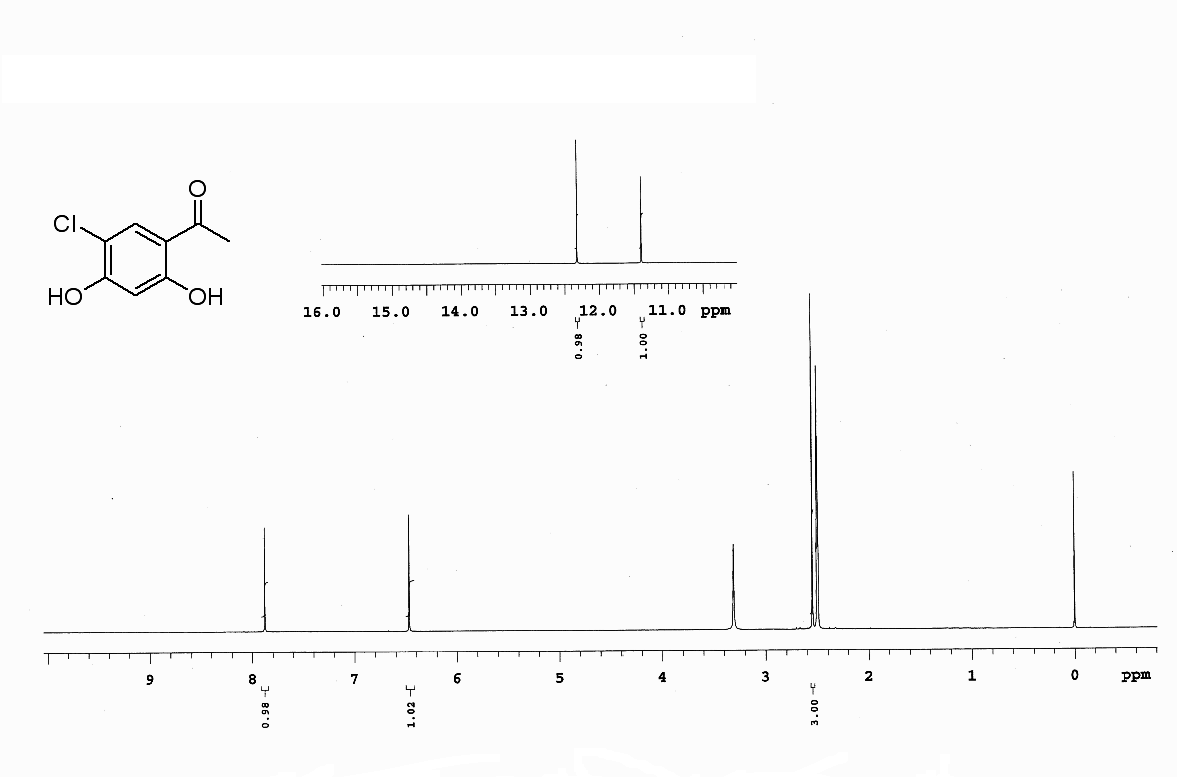


**Figure S1:** ^1^H NMR of compound **2**


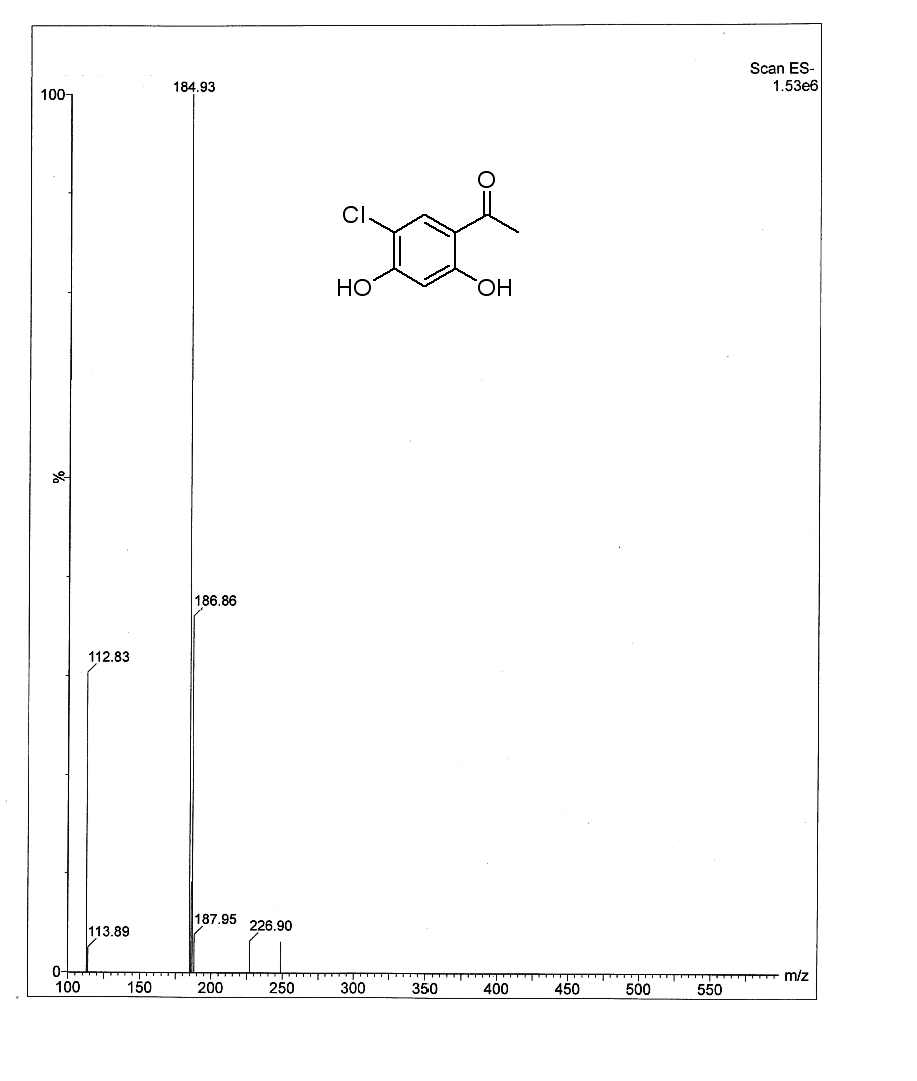


**Figure S2:** HRMS of compound **2**


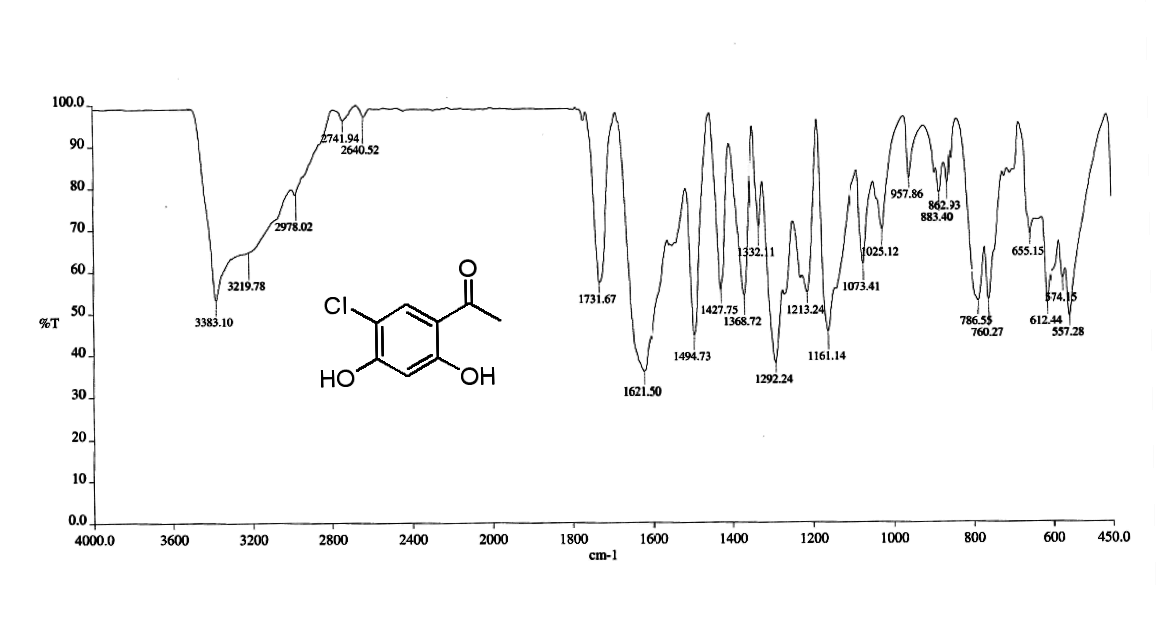


**Figure S3:** IR spectra of compound **2**


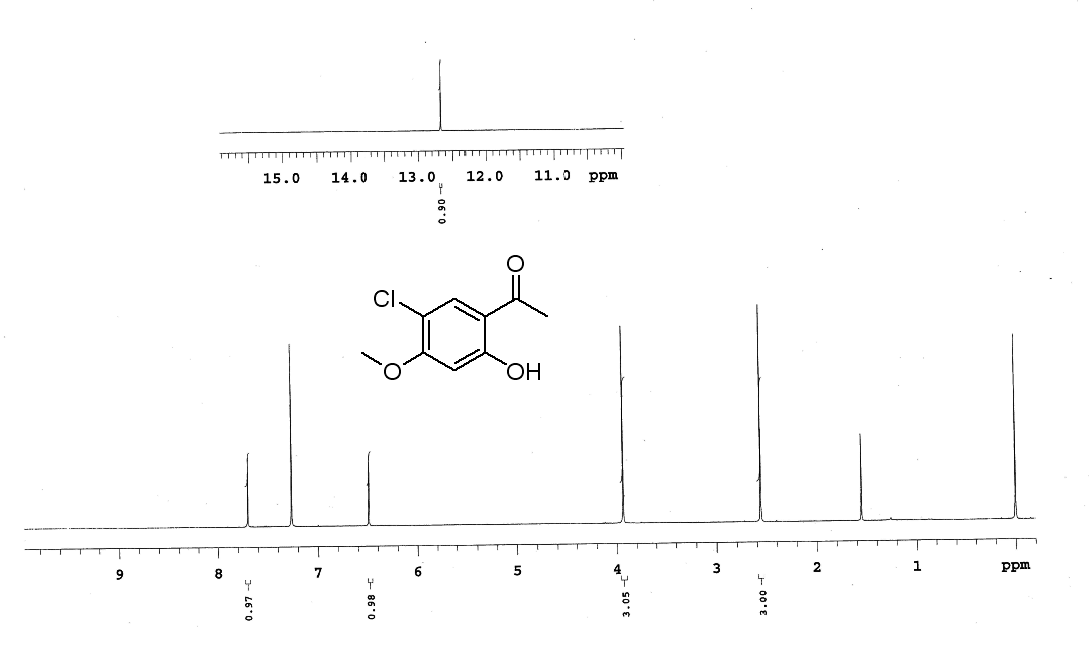


**Figure S4:** ^1^H NMR of compound **3**


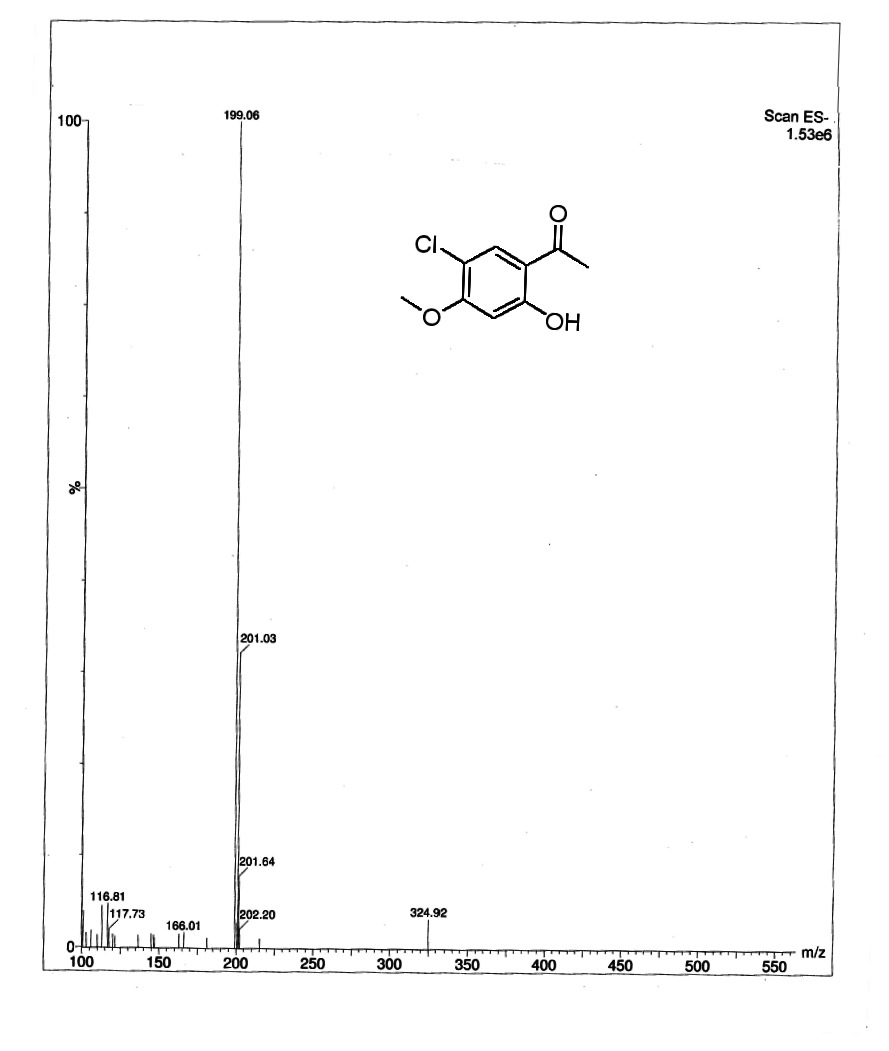


**Figure S5:** HRMS of compound **3**


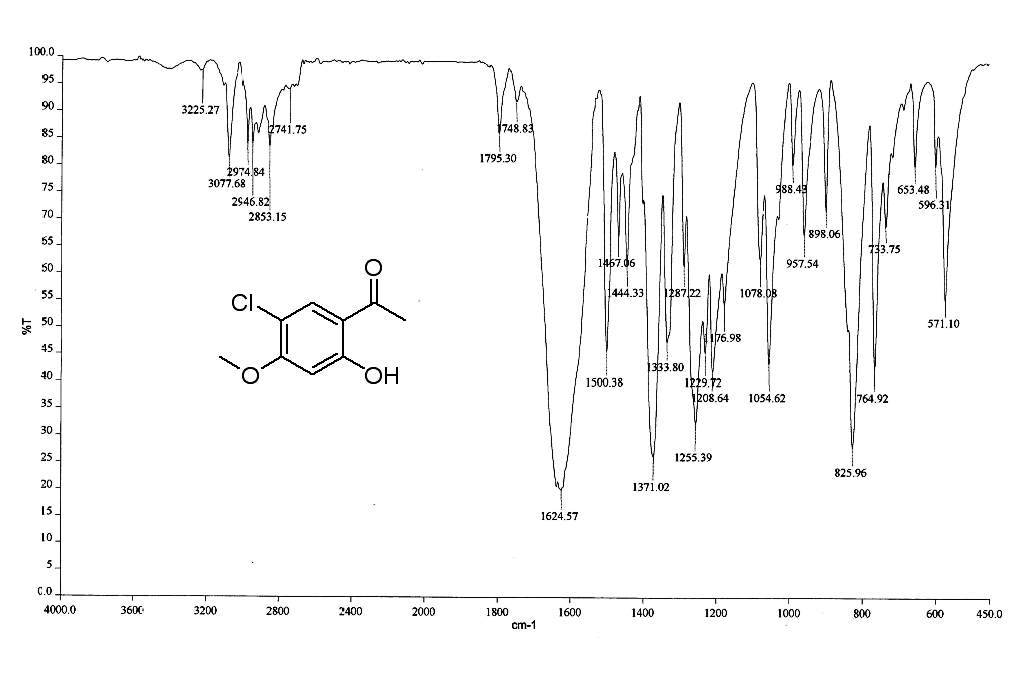


**Figure S6:** IR spectra of compound **3**


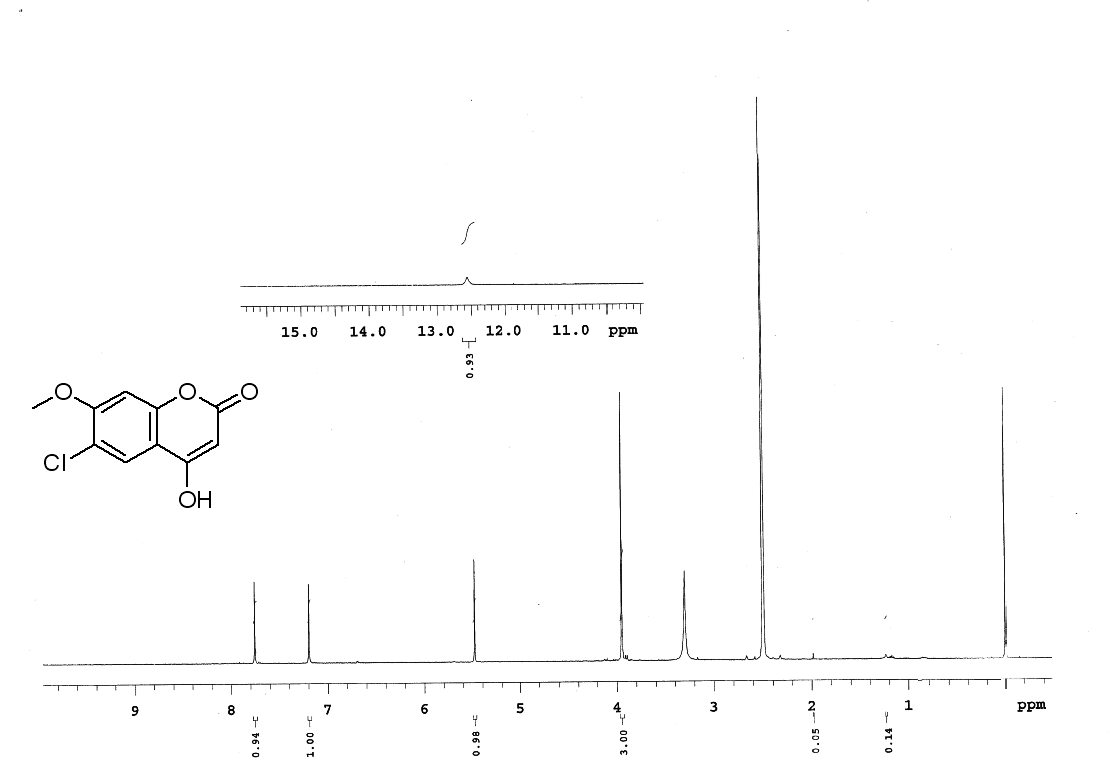


**Figure S7:** ^1^H NMR of compound **4**


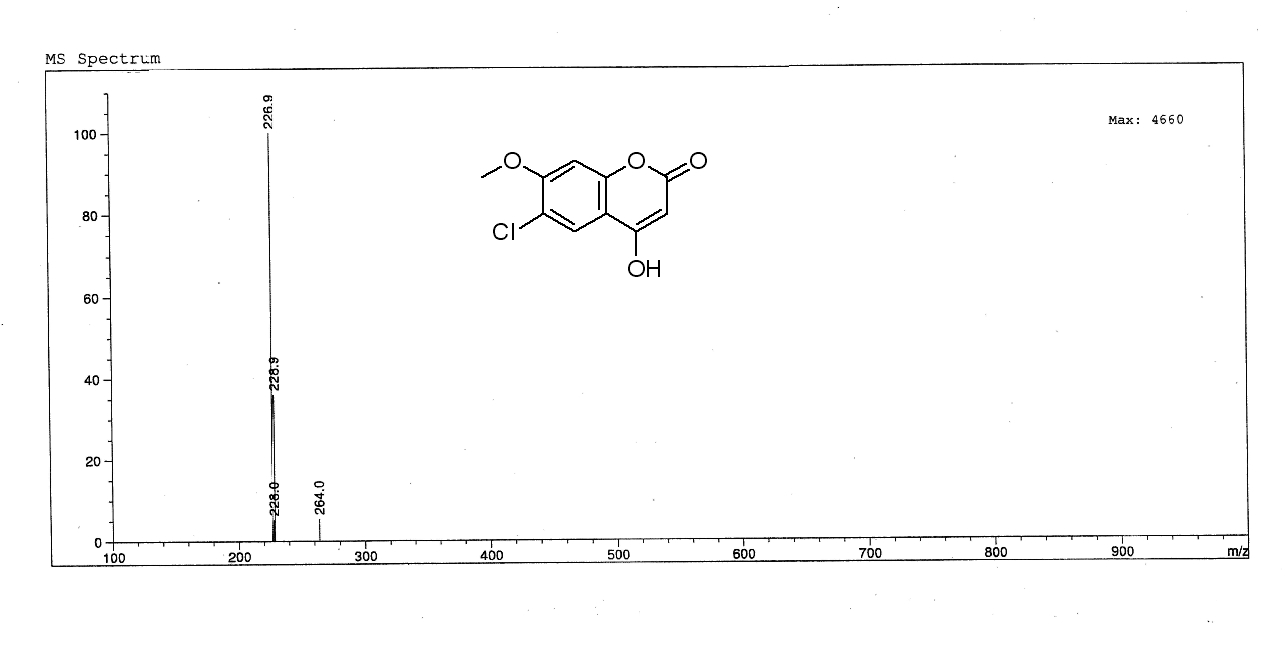


**Figure S8:** HRMS of compound **4**


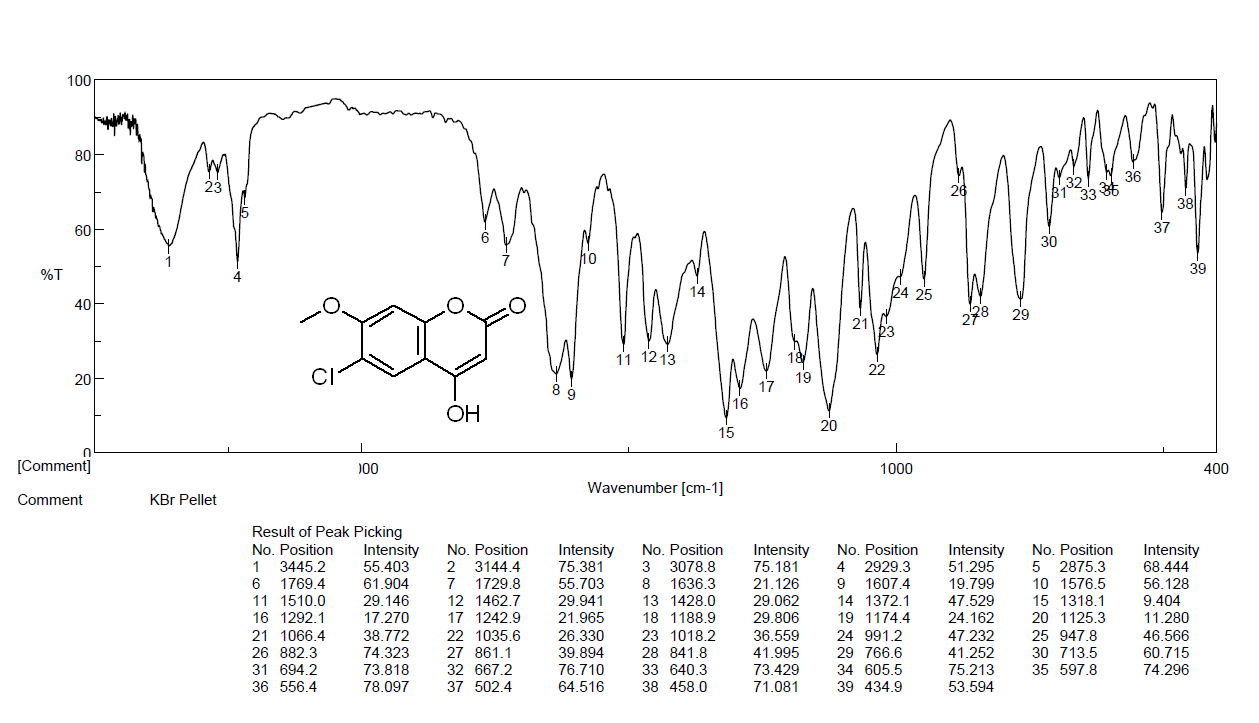


**Figure S9:**IR spectra of compound **4**


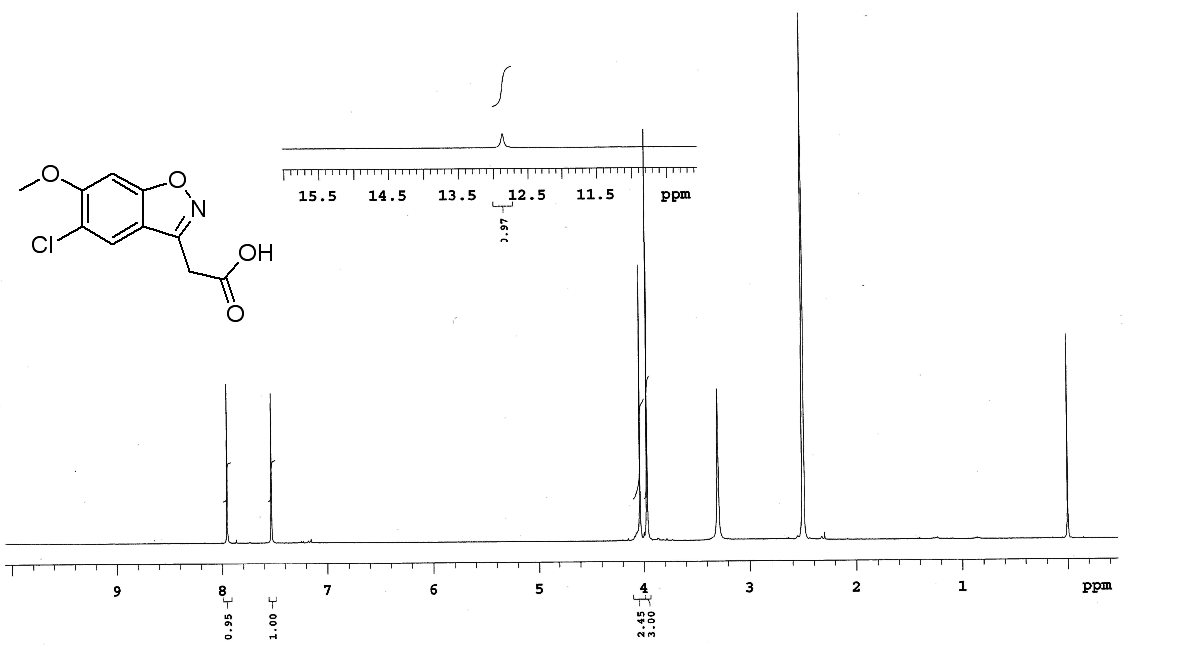


**Figure S10:** ^1^H NMR of compound **5**


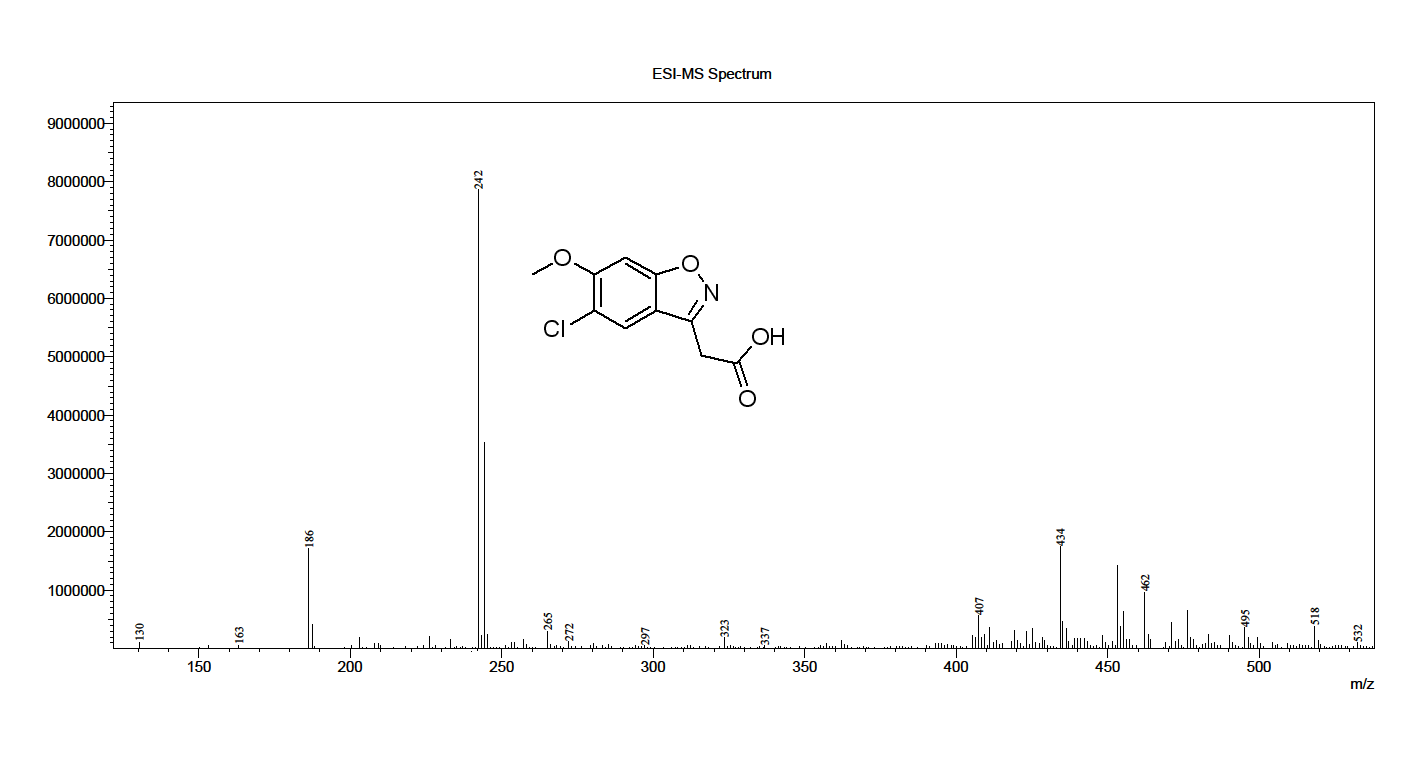


**Figure S11:** HRMS of compound **5**


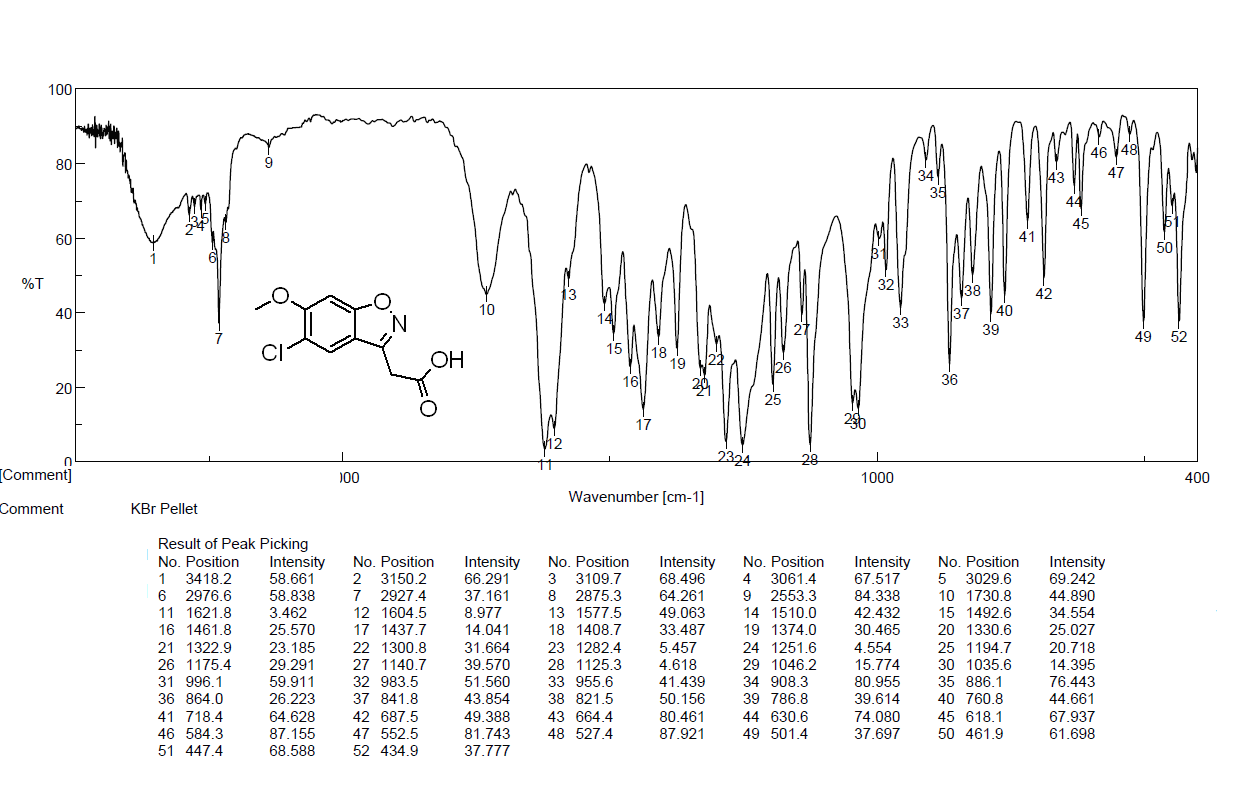


**Figure S12:** IR spectra of compound **5**


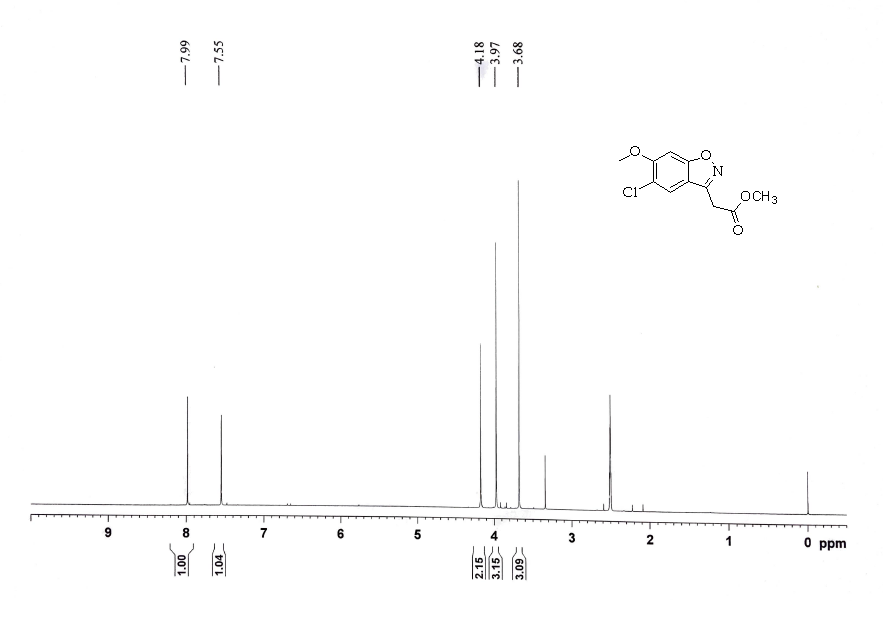


**Figure S13:** ^1^H NMR of compound **6**


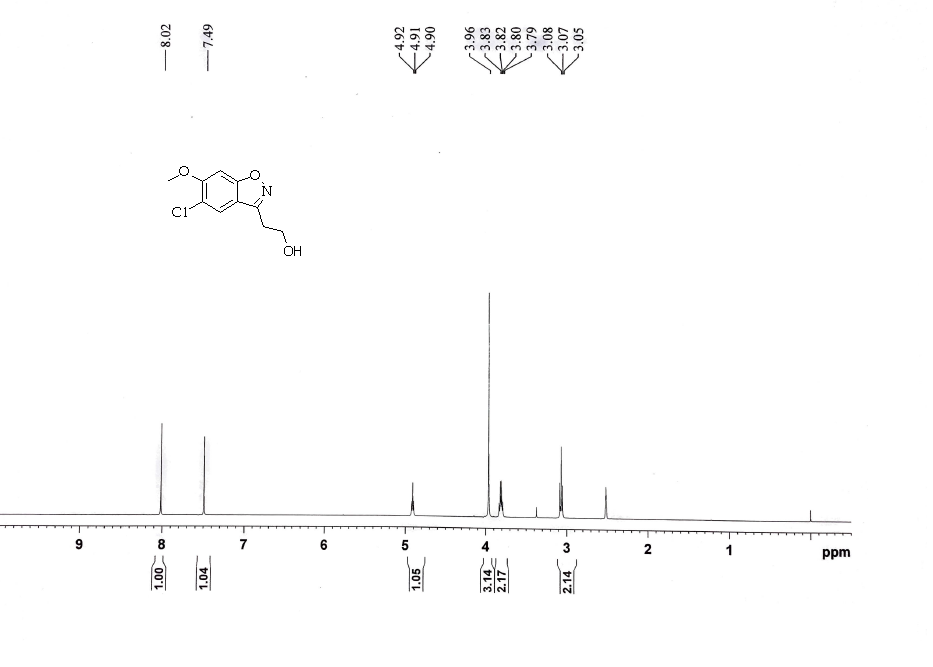


**Figure S14:** ^1^H NMR of compound **7**


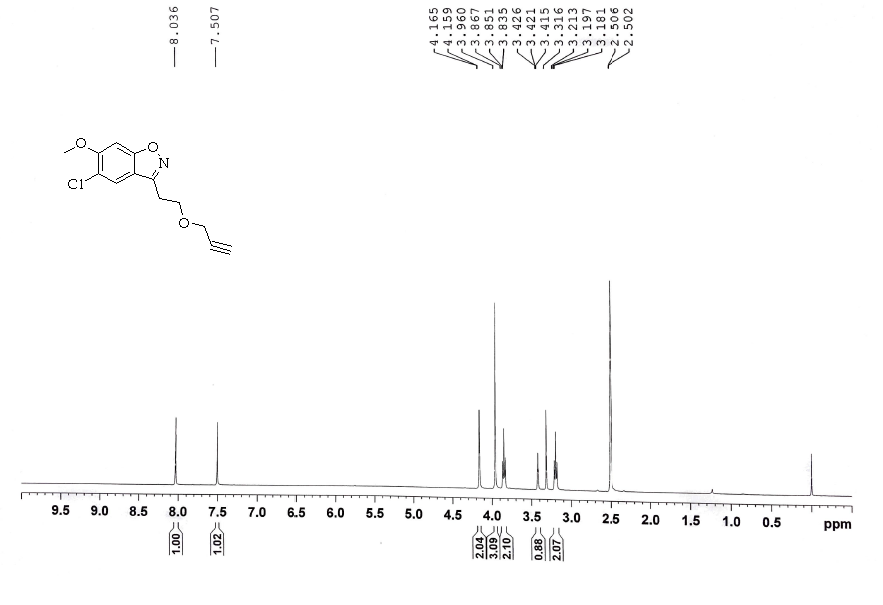


**Figure S15:** ^1^H NMR of compound **8**


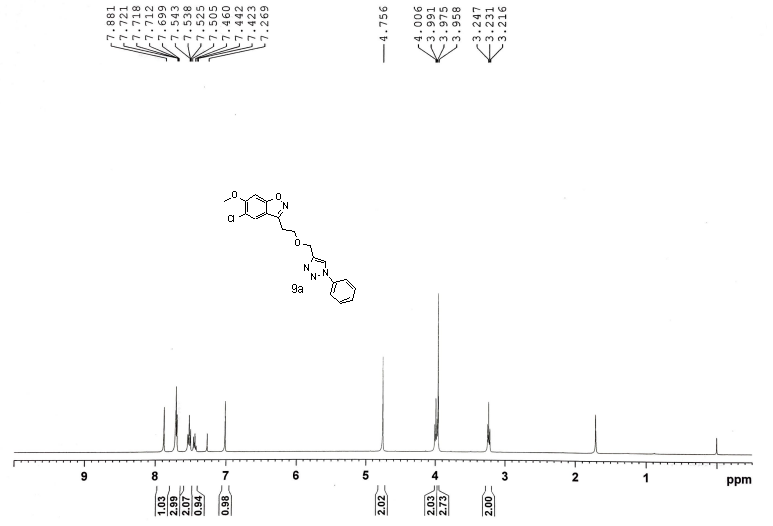


**Figure S16:** ^1^H NMR of compound **9a**


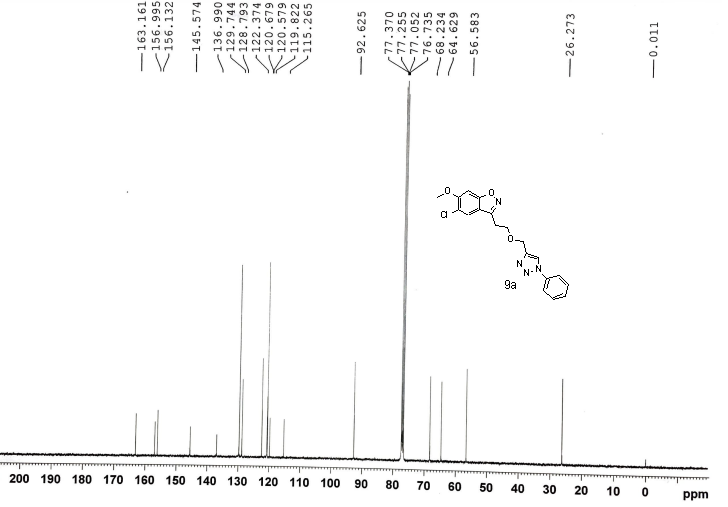


**Figure S17:** ^13^C NMR of compound **9a**


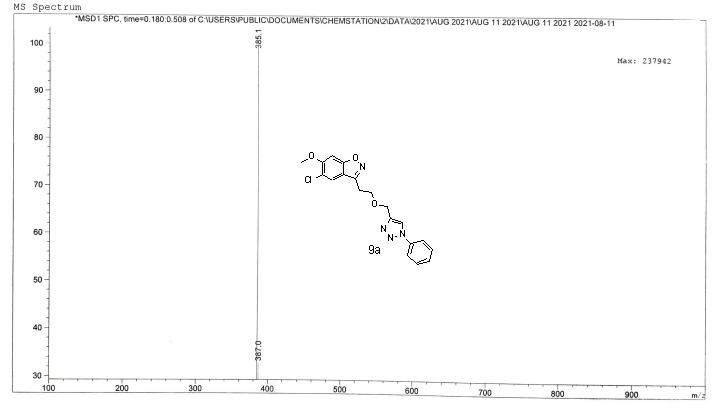


**Figure S18:** HRMS of compound **9a**

**Figure S19:** IR spectrum of compound **9a**


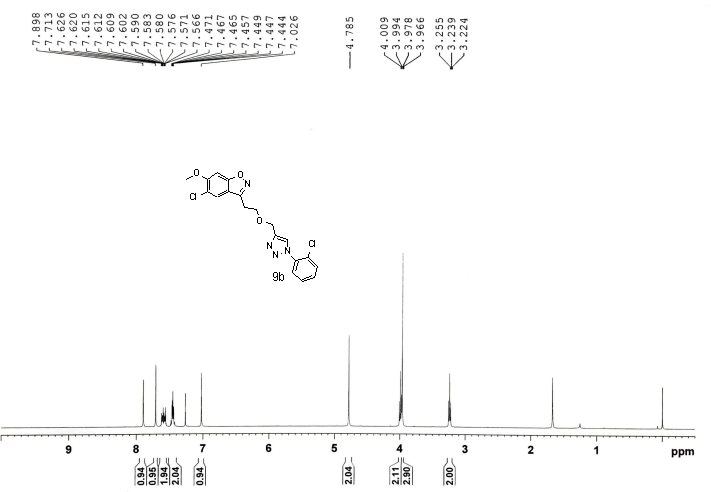


**Figure S20:** ^1^H NMR of compound **9b**


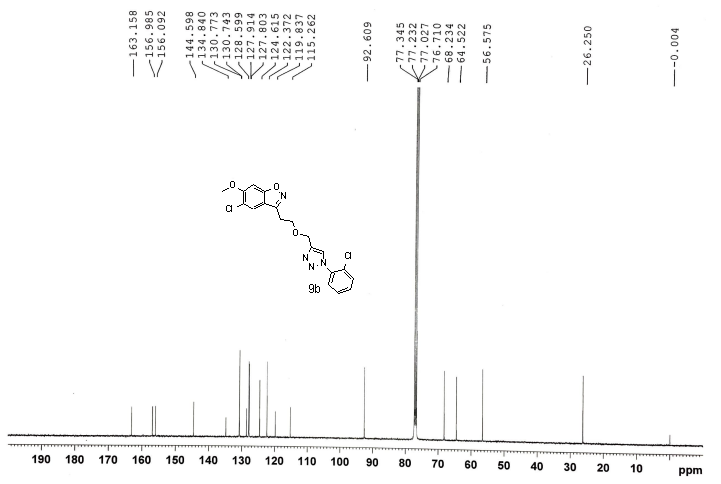


**Figure S21:** ^13^C NMR of compound **9b**


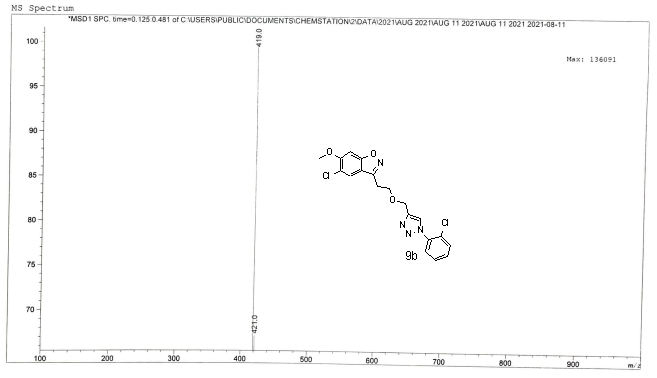


**Figure S22:** HRMS of compound **9b**

**Figure S23:** IR spectrum of compound **9b**


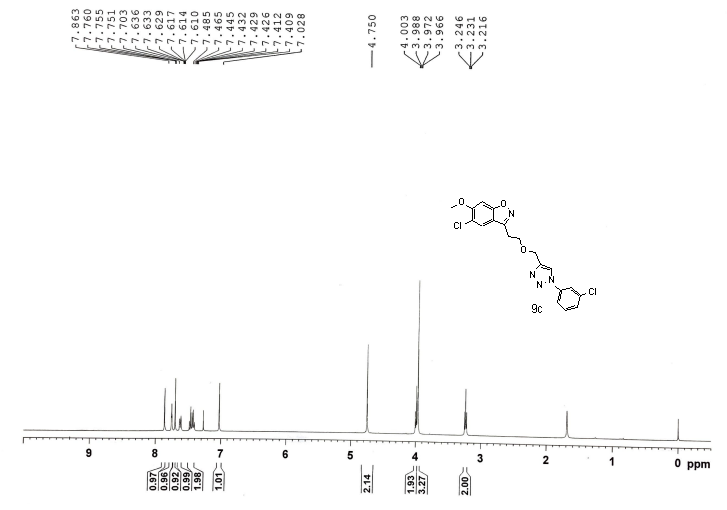


**Figure S24:** ^1^H NMR of compound **9c**


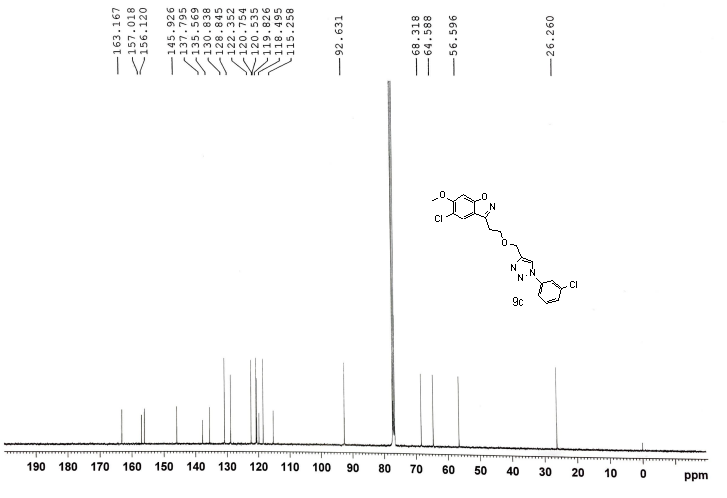


**Figure S25:** ^13^C NMR of compound **9c**


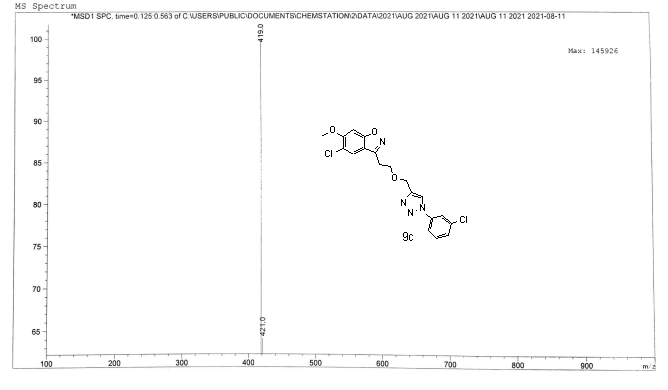


**Figure S26:** HRMS of compound **9c**

**Figure S27:** IR spectrum of compound **9c**


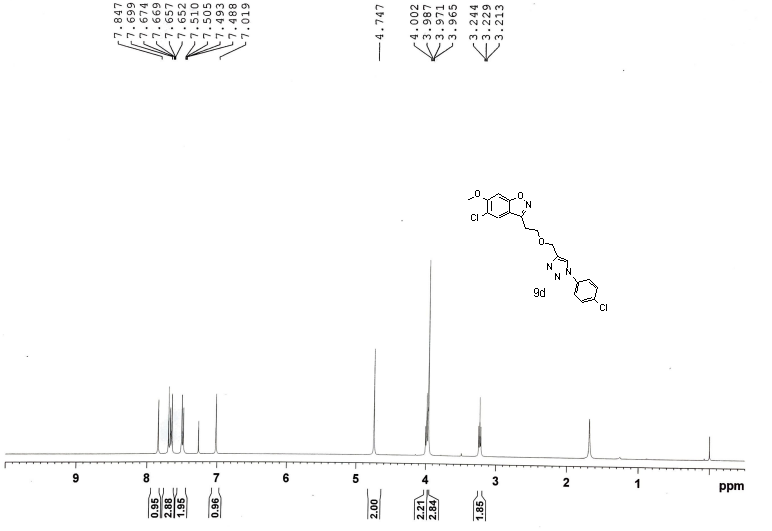


**Figure S28:** ^1^H NMR of compound **9d**


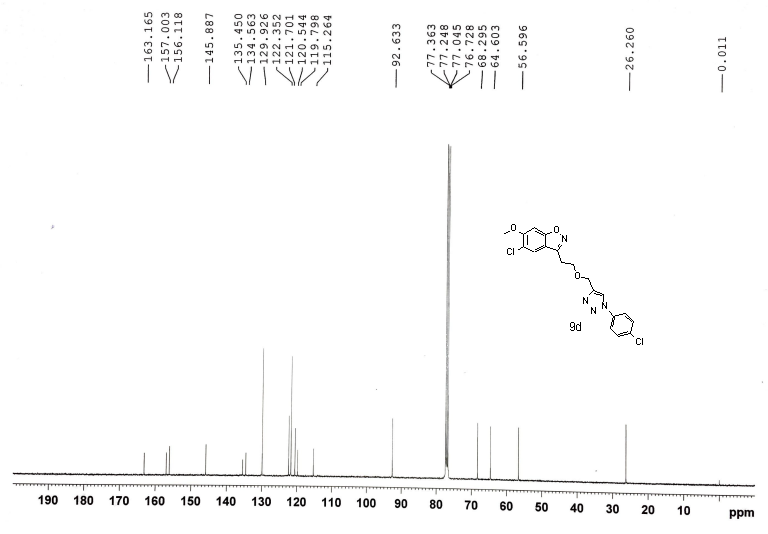


**Figure S29:** ^13^C NMR of compound **9d**


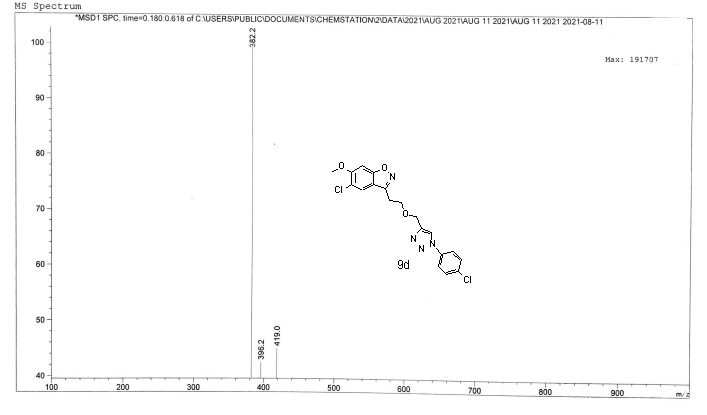


**Figure S30:** HRMS of compound **9d**

**Figure S31:** IR spectrum of compound **9d**


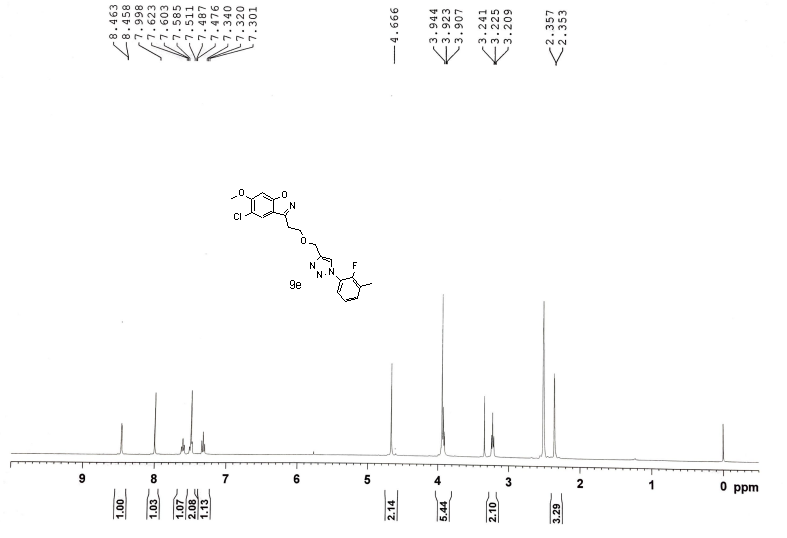


**Figure S32:** ^1^H NMR of compound **9e**


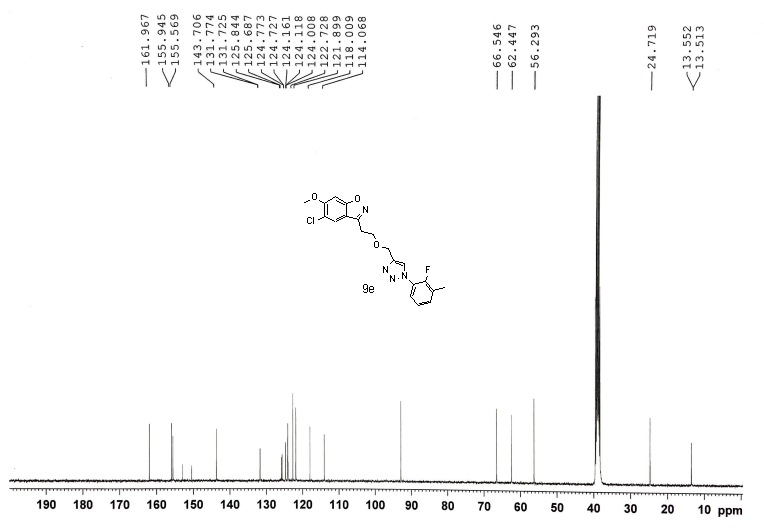


**Figure S33:** ^13^C NMR of compound **9e**


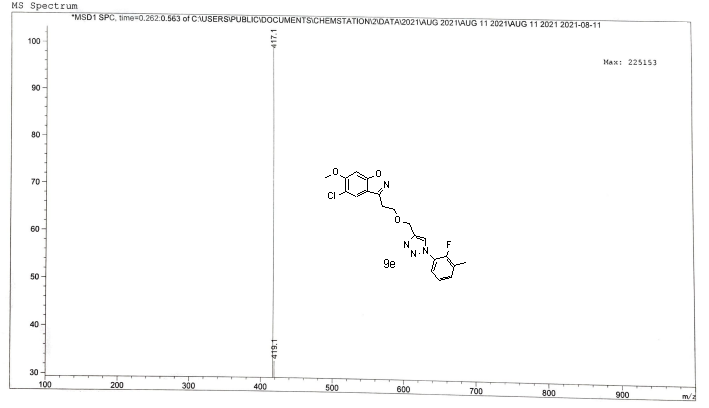


**Figure S34:** HRMS of compound **9e**

**Figure S35:** IR spectrum of compound **9e**


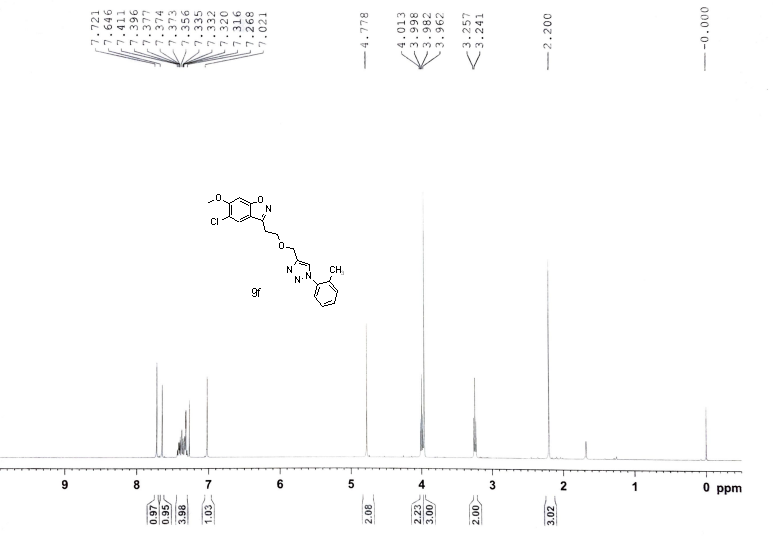


**Figure S36:** ^1^H NMR of compound **9f**


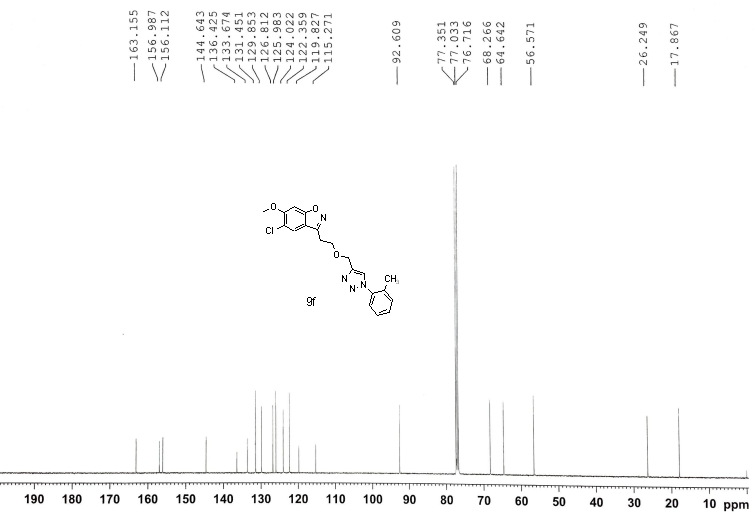


**Figure S37:** ^13^C NMR of compound **9f**


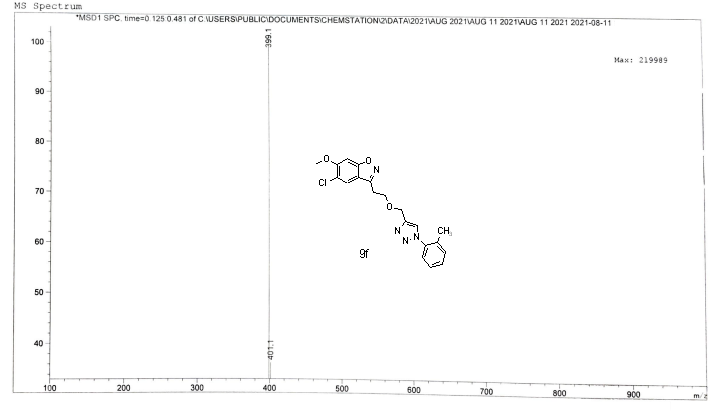


**Figure S38:** HRMS of compound **9f**

**Figure S39:** IR spectrum of compound **9**f


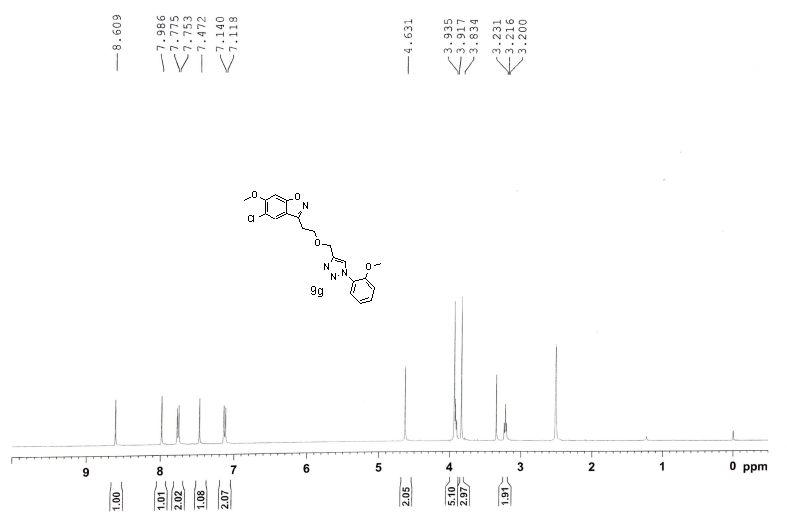


**Figure S40:** ^1^H NMR of compound **9g**


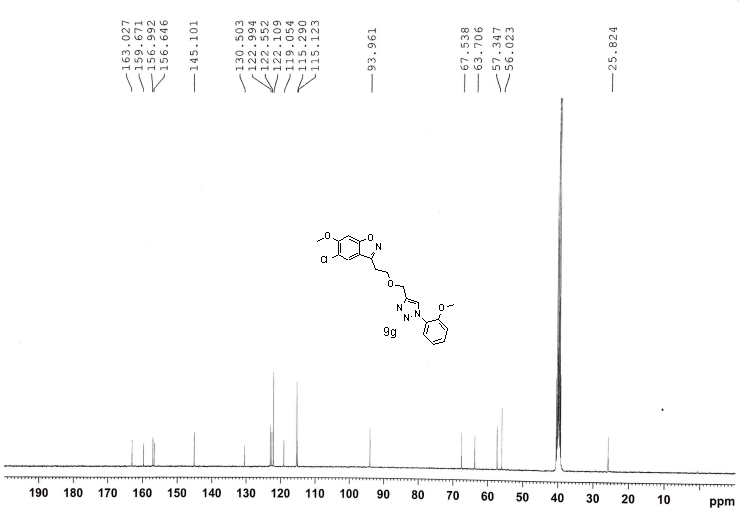


**Figure S41:** ^13^C NMR of compound **9g**


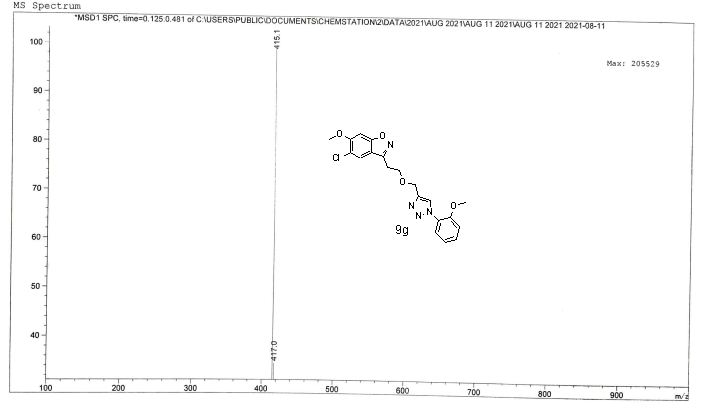


**Figure S42:** HRMS of compound **9g**

**Figure S43:** IR spectrum of compound **9g**


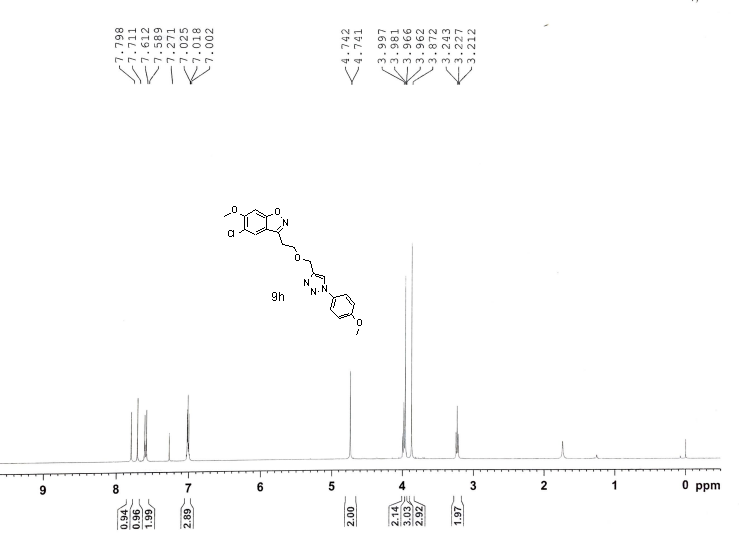


**Figure S44:** ^1^H NMR of compound **9h**
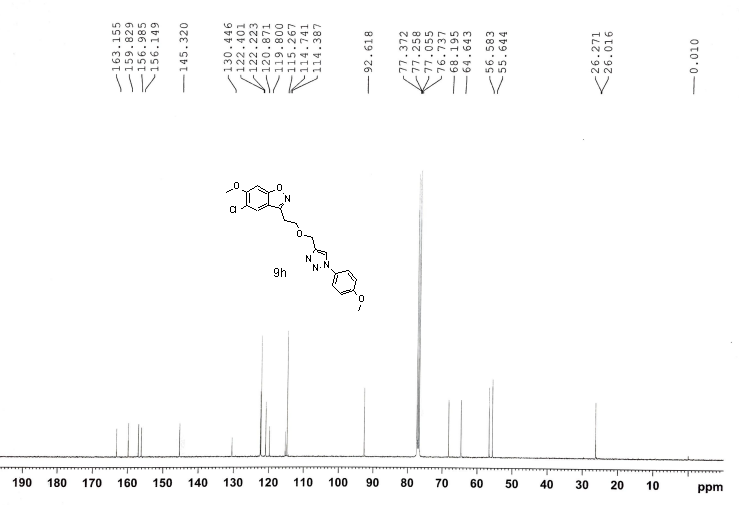


**Figure S45:** ^13^C NMR of compound **9h**


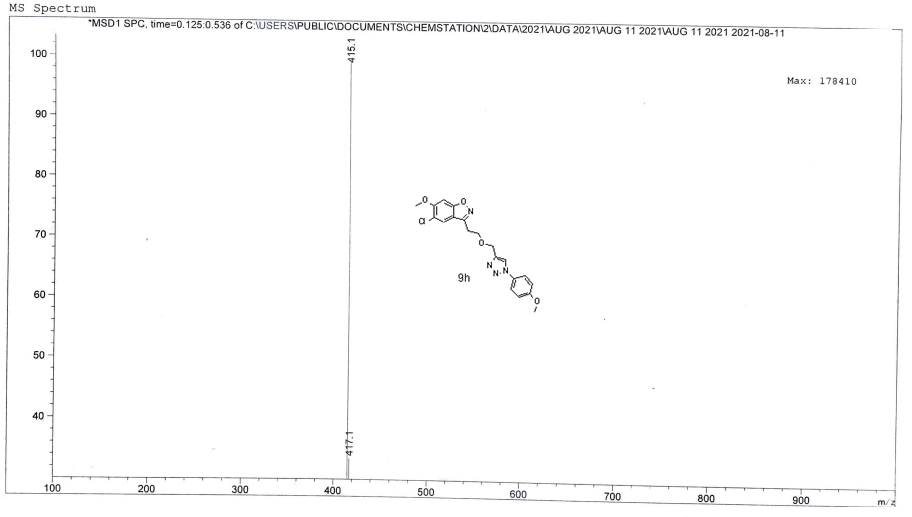


**Figure S46:** HRMS of compound **9h**

**Figure S47:** IR spectrum of compound **9h**


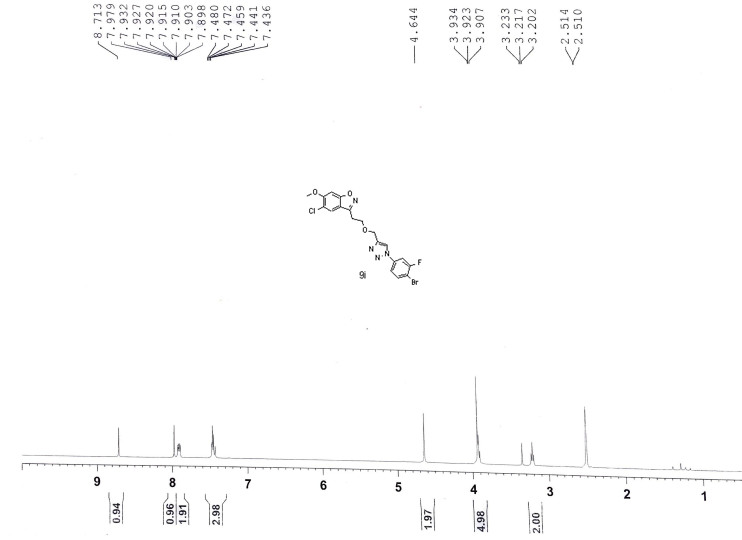


**Figure S48:** ^1^H NMR of compound **9i**


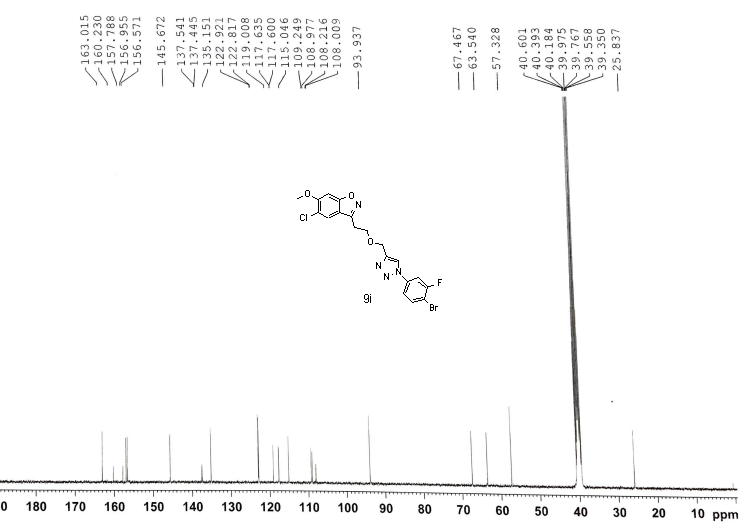


**Figure S49:** ^13^C NMR of compound **9i**


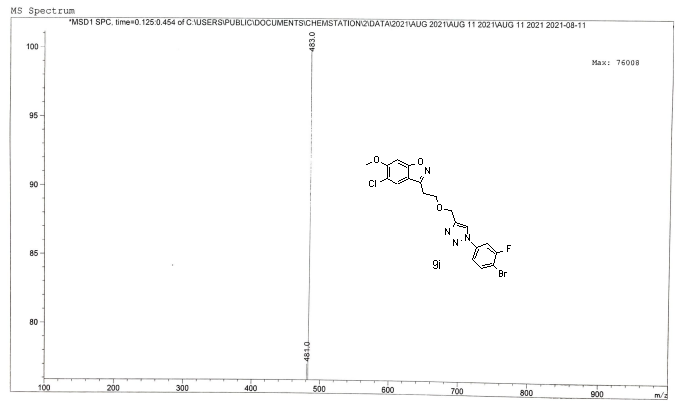


**Figure S50:** HRMS of compound **9i**

**Figure S51:** IR spectrum of compound **9i**


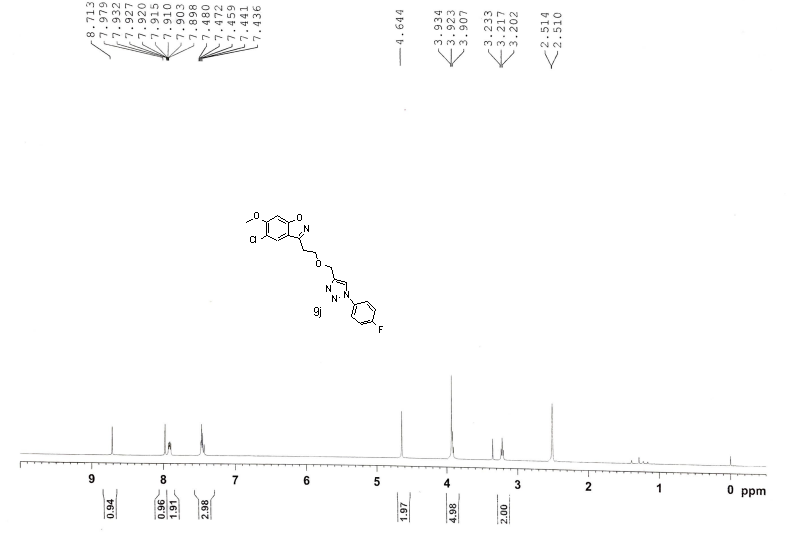


**Figure S52:** ^1^H NMR of compound **9j**


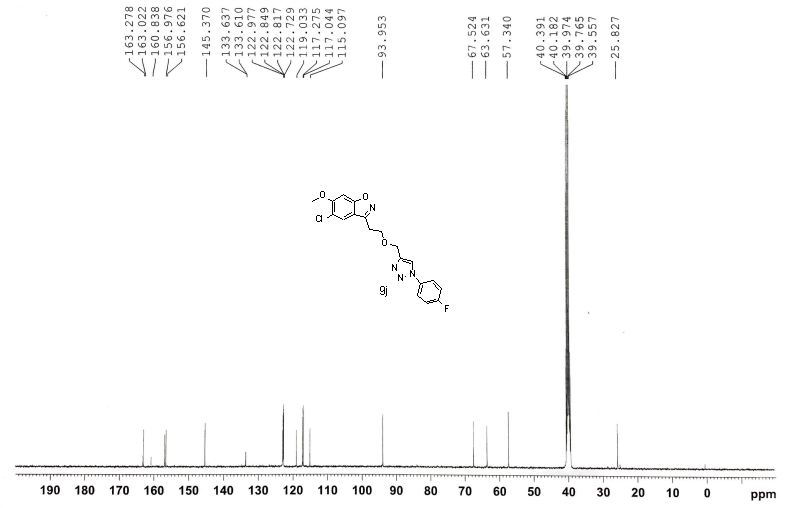


**Figure S53:** ^13^C NMR of compound **9j**


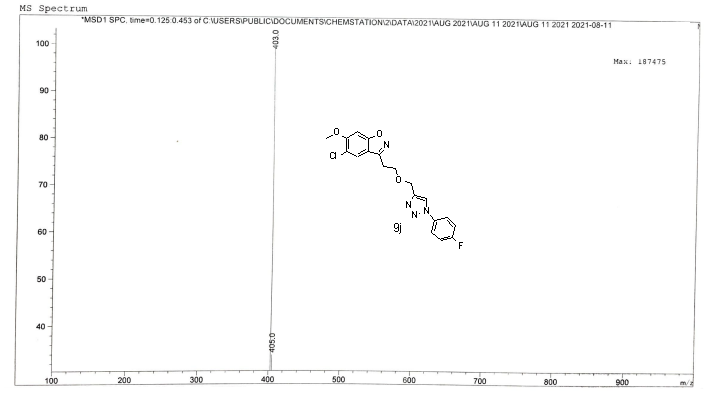


**Figure S54:** HRMS of compound **9j**

**Figure S55:** IR spectrum of compound **9j**


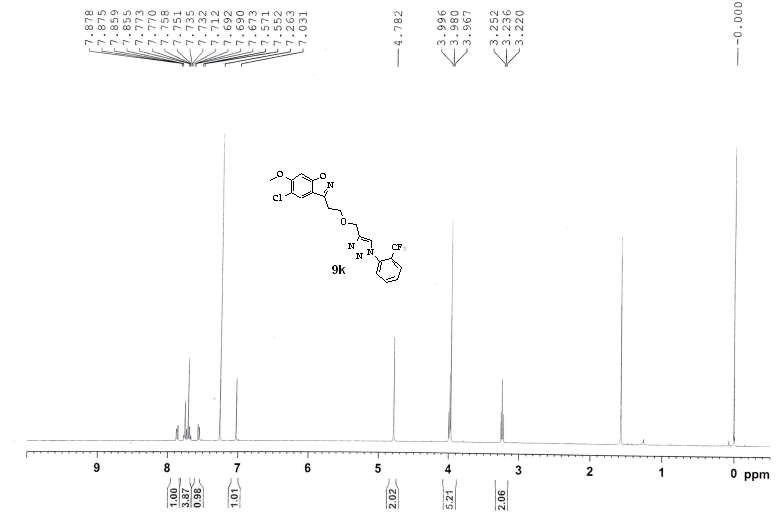


**Figure S56:** ^1^H NMR of compound **9k**


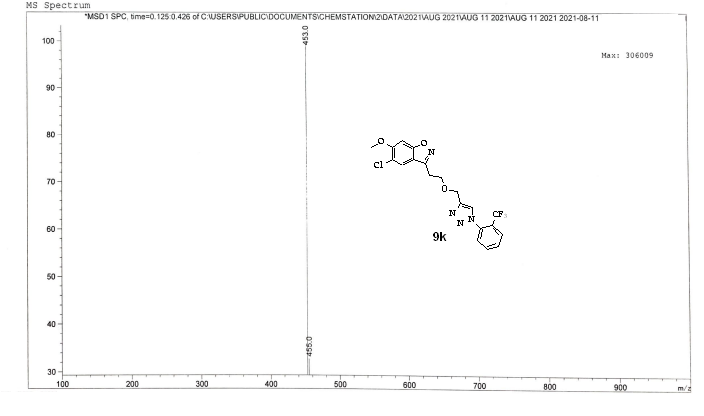


**Figure S57:** HRMS of compound **9k**

**Figure S58:** IR spectrum of compound **9k**


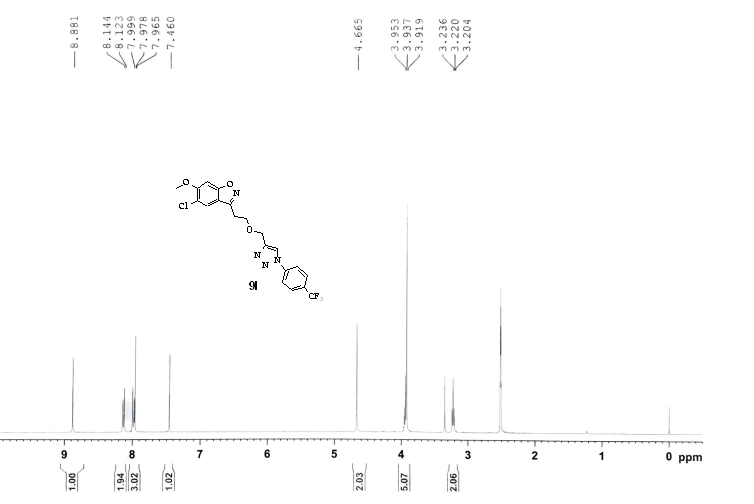


**Figure S59:** ^1^H NMR of compound **9l**


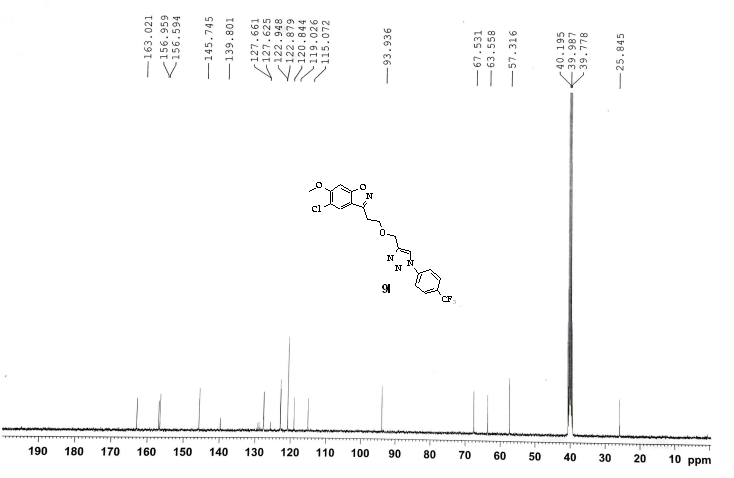


**Figure S60:** ^13^C NMR of compound **9l**


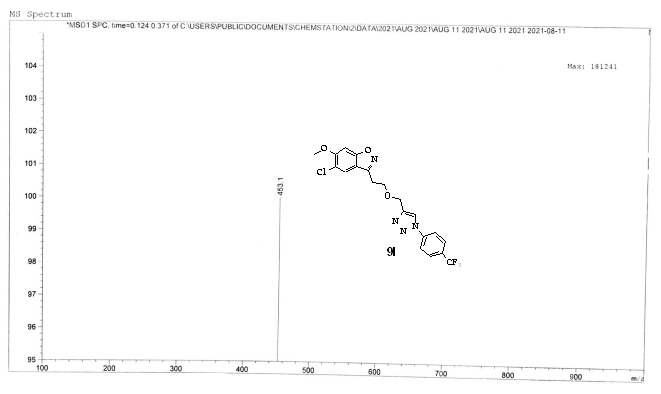


**Figure S61:** HRMS of compound **9l**

**Figure S62:** IR spectrum of compound **9l**


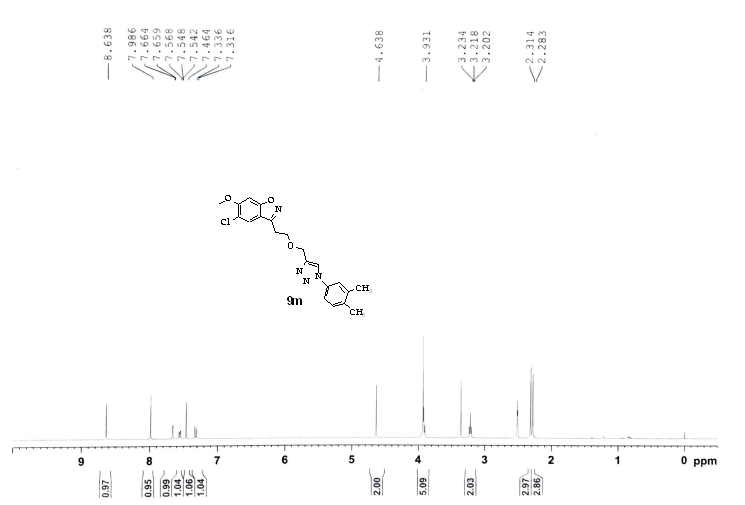


**Figure S63:** ^1^H NMR of compound **9m**


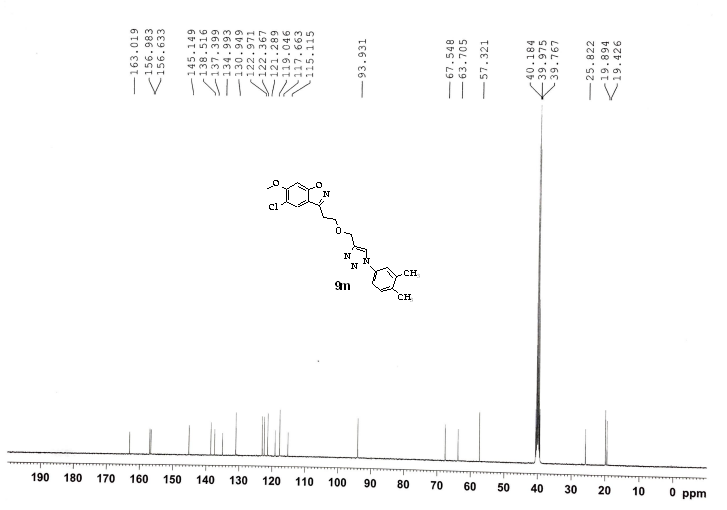


**Figure S64:** ^13^C NMR of compound **9m**


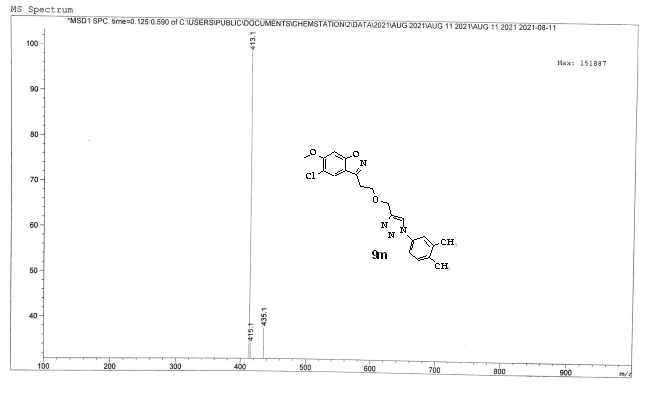


**Figure S65:** HRMS of compound **9m**

**Figure S66:** IR spectrum of compound **9m**


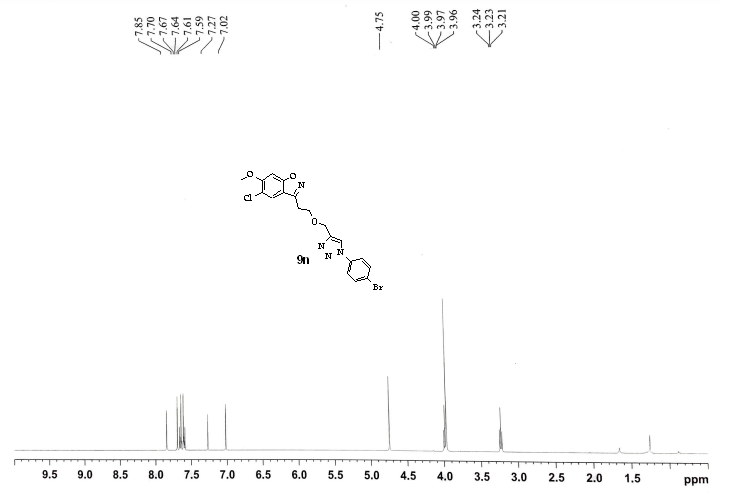


**Figure S67:** ^1^H NMR of compound **9n**


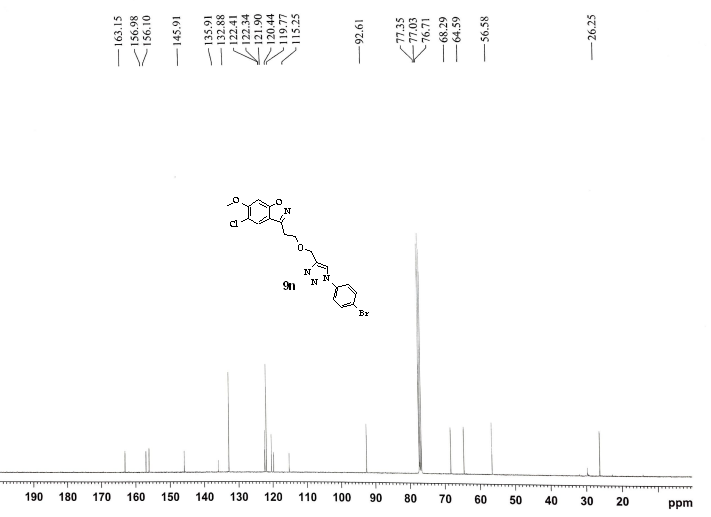


**Figure S68:** ^13^C NMR of compound **9n**


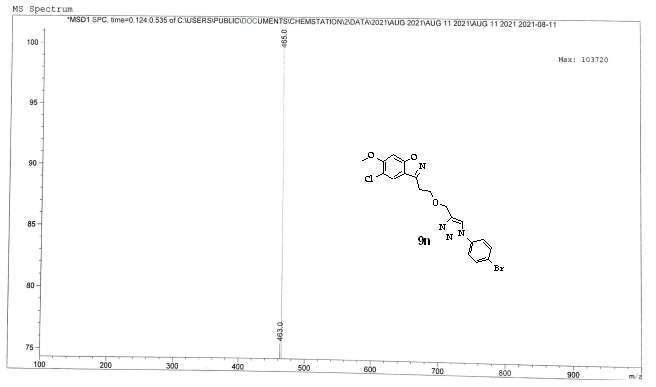


**Figure S69:** HRMS of compound **9n**

**Figure S70:** IR spectrum of compound **9n**


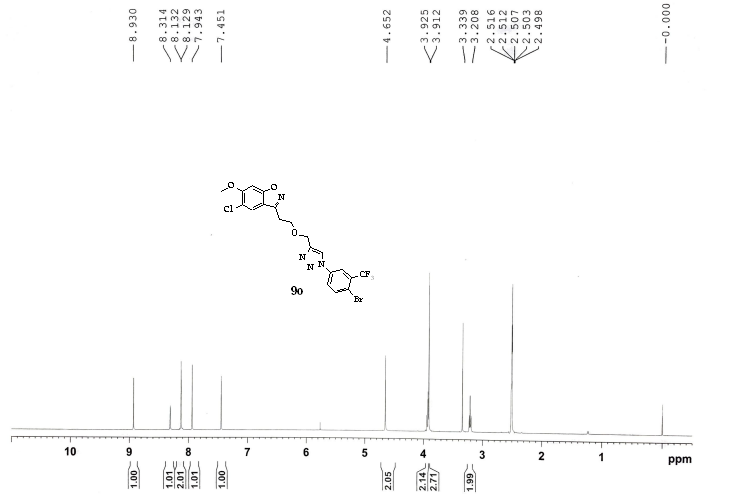


**Figure S71:** ^1^H NMR of compound **9o**


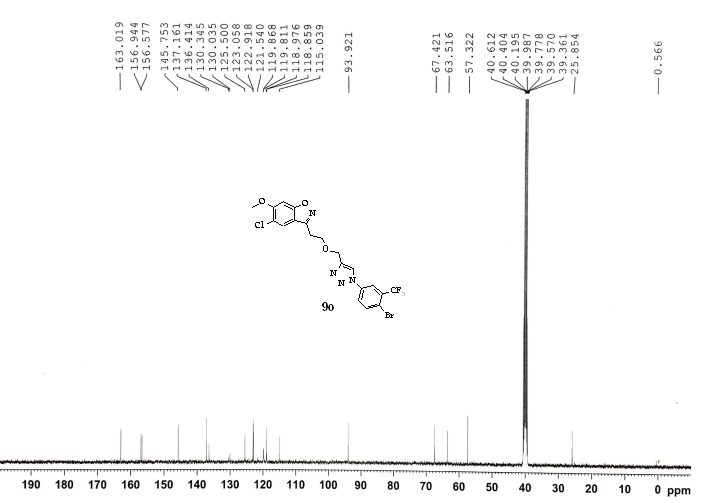


**Figure S72:** ^13^C NMR of compound **9o**


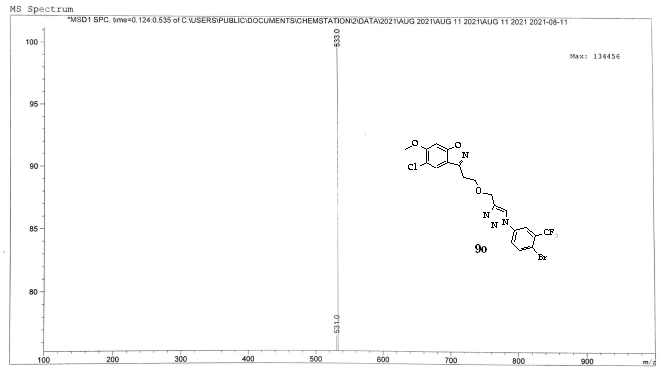


**Figure S73:** HRMS of compound **9o**

**Figure S74:** IR spectrum of compound **9o**
